# Linear-Scaling Asymmetric Triples Correction through the Solution of the DLPNO–CCSD Lambda Equations: DLPNO–CCSD(T)_Λ_

**DOI:** 10.1021/acs.jctc.6c00758

**Published:** 2026-07-21

**Authors:** Elizabeth C. Toth, Andy Jiang, Henry F. Schaefer

**Affiliations:** Center for Computational Quantum Chemistry, Department of Chemistry, 1355 University of Georgia, Athens, Georgia 30602, United States

## Abstract

In this research, we derive equations for solving for the stationary points of the DLPNO–CCSD Lagrangian, in the *t*_1_-transformed formalism introduced earlier and as currently implemented in the Psi4 quantum chemistry software package. These lambda equations in the local pair natural orbital basis allow for the evaluation of CCSD­(T)_Λ_ energetics with linear-scaling computational effort, also known as the asymmetric triples correction. This DLPNO–CCSD­(T)_Λ_ method allows for accurate triples contributions to be computed for larger molecules, especially in cases that CCSD­(T) is known to be insufficient, such as with multireference systems and bond-breaking systems. We showcase the accuracy of our code on reaction energies, barrier heights, and noncovalent interaction energies. Also showcased are the capabilities of our code by evaluating DLPNO–CCSD­(T)_Λ_ energetics on large noncovalent dimers up to 112 atoms, as well as a rhodium catalyst complex containing 66 atoms.

## Graphical abstract

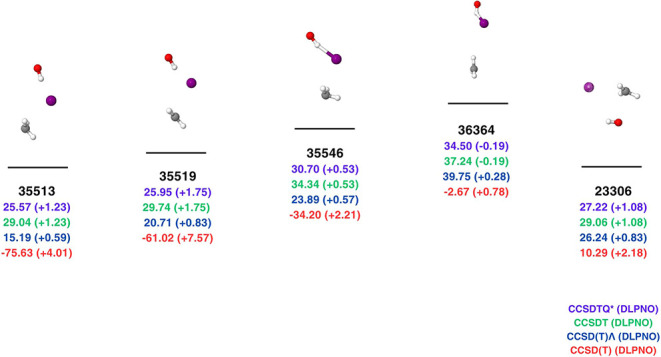

## I Introduction

Coupled-cluster theory (CC)^1,2^ has been pivotal in modern computational quantum chemistry, allowing for accurate theoretical models and predictions for molecular properties and other chemical phenomena within experimental error. Coupled-cluster provides for a size-extensive approximation to the full configuration interaction (FCI)^3,4^ energy and wave function at any level of truncation. The size-extensivity arises from the exponential ansatz

1 $$\left|\Psi_{\mathrm{CC}}\right\rangle =e^{T}\left|\Psi_{0}\right\rangle$$

2 $$E_{\mathrm{CC}}=\left\langle \Psi_{0}\right|e^{-T}Ĥe^{T}\left|\Psi_{0}\right\rangle$$

where |Ψ_0_⟩ represents a single reference Slater determinant derived from selecting o occupied orbitals out of a total of *N* basis functions in a finite basis set, typically derived using Hartree–Fock theory. The cluster operator *T* encapsulates the sum of all electronic excitations considered from the reference determinant. Through this exponential ansatz, the size-extensive property of coupled-cluster theory arises from higher order excitations being encapsulated by “disconnected” lower order excitations. For example, in CCSD (coupled cluster theory with singles and doubles), where *T* = *T*_1_ + *T*_2_, much of the effect of configuration interaction (CI) quadruples is encapsulated through the $\frac{1}{2}T_{2}^{2}$ that arises from e^*T*_1_+*T*_2_^. This gives coupled-cluster methods rapid convergence to the FCI limit, highlighted by excellent agreement with respect to reaction energies,^5–13^ equilibrium geometries and spectroscopic constants,^14–20^ as well as magnetic properties.^21–24^

In the coupled-cluster series of methods, the CCSD­(T)^25,26^ method has been dubbed the “gold standard” since it often gives results within “chemical accuracy” (1 kcal mol^–1^ from FCI relative energies).^11,27–31^ CCSD­(T) treats singles (*T*_1_) and doubles excitations (*T*_2_) at the CCSD level of theory, and then estimates triples excitations (*T*_3_) using converged CCSD singles and doubles amplitudes. This involves iterative $O\left(n^{6}\right)$ steps for CCSD as well as a noniterative $O\left(n^{7}\right)$ step for (*T*). In many cases, CCSD­(T) provides an excellent approximation to CCSDT (exact triples), which costs iterative $O\left(n^{8}\right)$ . However, there are many cases in which the (*T*) approximation is no longer reliable. This often arises in multireference cases and systems with stretched geometries.^32,33^ In these cases, the (*T*) correction produces unphysical values since the Hartree–Fock determinant is not a good reference.^34,35^ In addition, near equilibrium geometry, (*T*) appears to benefit from error cancellation between singles and doubles contributions.^35^ Evidence suggests that the inclusion of higher order excitations can account for multireference effects,^36–39^ though computational costs quickly become intractable. The inclusion of full triples excitations (CCSDT) is requisite to study the dissociation of single bonds and full quadruples excitations (CCSDTQ) for double bonds, though these methods have computational costs of iterative $O\left(n^{8}\right)$ and $O\left(n^{10}\right)$ , respectively. Some of the failures of traditional perturbative approaches such as (*T*) can be attributed by the non-Hermiticity of the coupled-cluster wave function

3 $$\left\langle \Psi_{0}\right|e^{-T}Ĥ\neq \left\langle \Psi_{0}\right|e^{-T}E_{\mathrm{CC}}$$

Through the application of Lagrange multipliers, via the $\hat{\Lambda }$ operator, a “left-handed” wave function can be derived.^40–44^ These $\hat{\Lambda }$ amplitudes are also known as “de-excitation” operators, and can be used to enforce the following constraint

4 $$\left\langle \Psi_{0}\right|e^{-T}Ĥ=\left\langle \Psi_{0}\right|\left(1+\mathrm{\Lambda ̂}\right)e^{-T}E_{\mathrm{CC}}$$

In canonical perturbation theories, it is assumed that the coupled-cluster wave function is approximately Hermitian (the assumption being *T*_1_ ≈ Λ_1_ and *T*_2_ ≈ Λ_2_), so the left-handed wave functions are not utilized in their derivation. Near equilibrium geometries, this approximation is valid and does not produce large errors. However, at stretched bond lengths or in systems with significant multireference character, this assumption does not hold, leading to a catastrophic failure of (*T*) at estimating the true energy contribution from triples excitations.^45^

With the incorporation of these $\hat{\Lambda }$ amplitudes, an alternative triples excitation can be formulated from perturbation theory that considers the left-handed wave function, called CCSD­(T)_Λ_.^34,46–51^ Through this formalism, CCSD­(T) can be viewed of as an approximation to CCSD­(T)_Λ_ in the limit that *T*_1_ = Λ_1_ and *T*_2_ = Λ_2_. In addition, CCSD­(T)_Λ_ is expected to generally give answers closer to CCSDT than CCSD­(T). Albeit, it is incorrect to state that CCSD­(T)_Λ_ is a direct approximation of CCSDT. Rather, this arises from the fact that both levels of theory are approximations of FCI that recover more Hermiticity than CCSD­(T).

Since CCSD­(T)_Λ_ is roughly a factor of 2 more expensive than CCSD­(T), and many applications of quantum chemistry are close to equilibrium geometries, CCSD­(T)_Λ_ has not been as widely available as CCSD­(T). However, in the realm of high-accuracy quantum chemistry, and in more rigorous benchmarking studies, CCSD­(T)_Λ_ represents a more reliable approximation to the FCI limit than CCSD­(T).^52^ Thus, it is certainly a useful method to have at this given Pauling point of speed and accuracy (1 kcal mol^–1^ and formal $O\left(n^{7}\right)$ cost), especially for applications involving significant static correlation or multireference character.^53^

The endeavor to reduce the computational cost of such methods has resulted in the development of “local correlation” approaches in quantum chemistry. These methods introduce controllable approximations to the coupled-cluster wave function or energy to reduce the computational scaling of these methods without compromising accuracy. Early approaches, such as through the work of Schütz and Werner, apply localized molecular orbitals (LMOs) to the occupied space and projected atomic orbitals (PAOs)^54^ to the virtual space, applying this methodology to the MP2,^55^ CCSD­(T),^56,57^ and even to CCSDT-1b methods,^58^ representing the earliest known attempt to apply local correlation to post-CCSD­(T) effects.

In later years, the development of cluster-in-molecule (CIM)^59–62^ approaches of Li and co-workers inspired the development of local natural orbital (LNO) based coupled-cluster methods of arbitrary order.^63–68^ Meanwhile, the work of Schütz and Werner inspired the development of local pair natural orbital (LPNO)-based approaches,^69–85^ especially the state-of-the-art domain-based local pair natural orbital (DLPNO) approach of Neese and co-workers applied up to CCSD­(T),^71,72,80^ and extended up to CCSDTQ^83–85^ in our prior work. These methods achieve asymptotic linear-scaling, allowing them to be applicable for larger molecules. The local approximations introduced are controllable and convergent to their canonical counterparts, and have shown to be accurate within 1 kcal mol^–1^ up to CCSD­(T),^71,72,77–82^ and 0.1–0.25 kcal mol^–1^ for higher-order coupled cluster methods, such as CCSDT/CCSDTQ.^68,83,85^

## II Research Strategy

In this research, we apply the domain-based local pair natural orbital (DLPNO)^71,72^ approach to the CCSD­(T)_Λ_ method, following prior work on employing the DLPNO methods to a variety of coupled-cluster methods up to full quadruples.^82–85^ We show that with sufficiently tight parameters, we can replicate the results of canonical CCSD­(T)_Λ_ methods within 0.2–1.0 kcal mol^–1^, with linear-scaling cost, following the development of our *t*_1_-transformed DLPNO–CCSD­(T) code in Psi4.^82^ Although not directly used to evaluate the (*T*) perturbation, the *t*_1_-transformation is applied to the CCSD equations to decrease memory and runtime, as well as reduce the mathematical complexity of the equations. Since the working equations for DLPNO–CCSD in Psi4 ^82^ are different than in ORCA,^71^ the equations we present for solving the DLPNO–CCSD lambda equations will be different than the ones presented in the prior work by Neese and co-workers.^86^ In addition, this work will represent, to the best of our knowledge, the first known application of local correlation to CCSD­(T)_Λ_. We show that our new DLPNO–CCSD­(T)_Λ_ algorithm reproduces answers much closer to the best estimated FCI limit (through DLPNO–CCSDTQ corrected with local correlation error from DLPNO–CCSDT^83,85^ ) than CCSD­(T) in cases that the latter two disagree significantly with an *S*_*N*_2 reaction potential energy surface. In addition, we evaluate our code on large noncovalent dimers in the L7 data set to compare DLPNO–CCSD­(T) and DLPNO–CCSD­(T)_Λ_ energetics, containing large conjugated dispersion-bound dimers such as coronene dimer, as well as minima and transition states of a large rhodium catalyst complex containing 66 atoms. With our DLPNO–CCSD­(T)_Λ_ algorithm being linear scaling, it is possible to approach the FCI limit at the complete basis set at a significantly reduced cost for situations in which CCSD­(T) fails, allowing for gold-standard energetics to be evaluated for such systems.

## III Notation

We use the following conventions to describe the indices of matrices and tensors appearing in this work, analogous to the notation used in prior research:^82,83^

- μ, ν, λ, σ: atomic orbitals; these range from 1 to *n*_bf_, the number of basis functions
- *i*, *j*, *k*, *l*: canonical and local occupied molecular orbitals; these range from 1 to *n*_occ_, the number of occupied orbitals
- *a*, *b*, *c*, *d*: canonical virtual molecular orbitals; these range from 1 to *n*_virt_, the number of virtual orbitals
- *p*, *q*, *r*, *s*: general canonical molecular orbitals; these range from 1 to *n*_occ_ + *n*_virt_
- μ̃, ν̃, λ̃, σ̃: projected atomic orbitals; these range from 1 to *n*_bf_
- μ̃_*ij*_, ν̃_*ij*_, λ̃_*ij*_, σ̃_*ij*_: projected atomic orbitals localized to pair *ij*; these range from 1 to *n*_pao,*ij*_, number of PAOs local to LMO pair *ij*
- μ̃_*ijk*_, ν̃_*ijk*_, λ̃_*ijk*_, σ̃_*ijk*_: projected atomic orbitals localized to triple *ijk*; these range from 1 to *n*_pao,*ijk*_, number of PAOs local to LMO triple *ijk*
- *k*_*ij*_, *l*_*ij*_, *m*_*ij*_, *n*_*ij*_: “Interacting” local molecular orbitals in a pair domain *ij*, defined as the set of all occupied orbitals *k* such that *ik* and *jk* forms a valid pair; these range from 1 to *n*_lmo, *ij*_, number of LMOs in the domain of LMO pair *ij*
- *a*_*ij*_, *b*_*ij*_, *c*_*ij*_, *d*_*ij*_: Pair natural orbitals in each pair domain *ij*; these range from 1 to *n*_pno, *ij*_, number of PNOs in the domain of LMO pair *ij*
- *l*_*ijk*_, *m*_*ijk*_, *n*_*ijk*_: “Interacting” local molecular orbitals in a triples domain *ijk*, defined as the set of all occupied orbitals *l* such that *il*, *jl*, and *kl* are all strong or weak pairs; these range from 1 to *n*_lmo, *ijk*_, number of LMOs in the domain of LMO triplet *ijk*
- *a*_*ijk*_, *b*_*ijk*_, *c*_*ijk*_, *d*_*ijk*_: Triples natural orbitals in each triples domain *ijk*; these range from 1 to *n*_tno, *ijk*_, number of TNOs in the domain of LMO triplet *ijk*
- *P*, *Q*: auxiliary basis functions for density-fitted ERIs; these range from 1 to *n*_aux_, number of auxiliary basis functions for density fitting
- *P*_*ij*_, *Q*_*ij*_: local auxiliary basis functions in each pair domain *ij*; these range from 1 to *n*_aux, *ij*_, number of auxiliary basis functions local to LMO pair *ij*
- *P*_*ijk*_, *Q*_*ijk*_: local auxiliary basis functions in each triples domain *ijk*; these range from 1 to *n*_aux, *ijk*_, number of auxiliary basis functions local to LMO triplet *ijk*

The relative sizes of these indices are usually

5 $$n_{\mathrm{pno},\mathrm{ij}}<n_{\mathrm{tno},\mathrm{ijk}}<n_{\mathrm{lmo},\mathrm{ij}}<n_{\mathrm{lmo},\mathrm{ijk}}\ll n_{\mathrm{pao},\mathrm{ij}}<n_{\mathrm{pao},\mathrm{ijk}}<<n_{\mathrm{aux},\mathrm{ij}}<n_{\mathrm{aux},\mathrm{ijk}}∼O\left(1\right)$$

$$n_{\mathrm{occ}}\ll n_{\mathrm{virt}}<n_{\mathrm{bf}}<n_{\mathrm{aux}}∼O\left(N\right)$$ 6

where *N* is the system size represented by the number of atoms or electrons.

## IV Theory

### IV.A Lagrangian (Lambda) Formalism

When a set of Langrange multipliers is introduced to the CCSD energy functional, we introduce a functional

7 $$L_{\mathrm{CC}}=\left\langle \Psi_{0}\right|\left(1+\Lambda \right)e^{-T}Ĥe^{T}\left|\Psi_{0}\right\rangle$$

In this expression, *Ĥ* represents the Hamiltonian and Λ is known as the “de-excitation” operator. At the CCSD level of theory, Λ = Λ_1_ + Λ_2_, consisting of the lambda amplitudes/Lagrange multipliers

8 $$\Lambda_{1}=\sum_{i,a}\lambda_{i}^{a}{â}_{i}^{†}{â}_{a}$$

9 $$\Lambda_{2}=\sum_{i,j,a,b}\lambda_{\mathrm{ab}}^{\mathrm{ij}}{â}_{i}^{†}{â}_{j}^{†}{â}_{b}{â}_{a}$$

λ_*i*_^*a*^ and λ_*ij*_^*ab*^ represent the singles and doubles “de-excitation” amplitudes. By expanding out eq 7, the expression becomes

10 $$L_{\mathrm{CCSD}}=\left\langle \Psi_{0}\right|\left(1+\Lambda_{1}+\Lambda_{2}\right)e^{-T}Ĥe^{T}\left|\Psi_{0}\right\rangle =\left\langle \Psi_{0}\right|e^{-T}Ĥe^{T}\left|\Psi_{0}\right\rangle +\left\langle \Psi_{0}\right|\Lambda_{1}e^{-T}Ĥe^{T}\left|\Psi_{0}\right\rangle +\left\langle \Psi_{0}\right|\Lambda_{2}e^{-T}Ĥe^{T}\left|\Psi_{0}\right\rangle$$

This becomes, by using the Λ_1_ and Λ_2_ operators on the left-hand side

11 $$L_{\mathrm{CCSD}}=\left\langle \Psi_{0}\right|e^{-T}Ĥe^{T}\left|\Psi_{0}\right\rangle +\sum_{i,a}\lambda_{i}^{a}\left\langle \Psi_{i}^{a}\right|e^{-T}Ĥe^{T}\left|\Psi_{0}\right\rangle +\sum_{i,j,a,b}\lambda_{\mathrm{ij}}^{\mathrm{ab}}\left\langle \Psi_{\mathrm{ij}}^{\mathrm{ab}}\right|e^{-T}Ĥe^{T}\left|\Psi_{0}\right\rangle$$

Overall, this becomes

12 $$L_{\mathrm{CCSD}}=E_{\mathrm{CCSD}}+\sum_{i,a}\lambda_{i}^{a}R_{i}^{a}+\sum_{i,j,a,b}\lambda_{\mathrm{ij}}^{\mathrm{ab}}R_{\mathrm{ij}}^{\mathrm{ab}}$$

From this expression, a constrained optimization can be utilized to find λ_*i*_^*a*^ and λ_*ij*_^*ab*^ through the residuals $l_{i}^{a}=\frac{\partial L}{\partial T_{i}^{a}}$ and $l_{\mathrm{ij}}^{\mathrm{ab}}=\frac{\partial L}{\partial T_{\mathrm{ij}}^{\mathrm{ab}}}$ . λ_*i*_^*a*^ and λ_*ij*_^*ab*^ are then iteratively solved for through

13 $$\lambda_{i}^{a}=\lambda_{i}^{a}-\frac{l_{i}^{a}}{\epsilon_{a}-\epsilon_{i}}$$

14 $$\lambda_{\mathrm{ij}}^{\mathrm{ab}}=\lambda_{\mathrm{ij}}^{\mathrm{ab}}-\frac{l_{\mathrm{ij}}^{\mathrm{ab}}}{\epsilon_{a}+\epsilon_{b}-\epsilon_{i}-\epsilon_{j}}$$

### IV.B (*T*)_Λ_ Energy Functional

A general equation for the perturbative triples correction can be written as^50^

15 $$E=\left\langle \Psi_{0}\right|\left(\Lambda_{1}+\Lambda_{2}\right)WT_{3}\left|\Psi_{0}\right\rangle$$

where *W* is the two-electron component of the Hamiltonian operator. With the (*T*) correction,^25^ Λ_1_ and Λ_2_ are approximated with *T*_1_^†^ and *T*_2_^†^. This expression forms the basis of the gold-standard (*T*) approximation

16 $$E_{\left(T\right)}=T_{\mathrm{ijk}}^{\mathrm{abc}}\cdot \left(\frac{4}{3}V_{\mathrm{ijk}}^{\mathrm{abc}}-2V_{\mathrm{ijk}}^{\mathrm{cba}}+\frac{2}{3}V_{\mathrm{ijk}}^{\mathrm{cab}}\right)$$

where the triples amplitude *T*_*ijk*_^*abc*^ is defined as

17 $$T_{\mathrm{ijk}}^{\mathrm{abc}}=\frac{W_{\mathrm{ijk}}^{\mathrm{abc}}}{\epsilon_{i}+\epsilon_{j}+\epsilon_{k}-\epsilon_{a}-\epsilon_{b}-\epsilon_{c}}$$

18 $$W_{\mathrm{ijk}}^{\mathrm{abc}}=P_{L}\left[\left(\mathrm{ia}|\mathrm{bd}\right)t_{\mathrm{kj}}^{\mathrm{cd}}-\left(\mathrm{ia}|\mathrm{jl}\right)t_{\mathrm{kl}}^{\mathrm{cb}}\right]$$

and

$$V_{\mathrm{ijk}}^{\mathrm{abc}}=W_{\mathrm{ijk}}^{\mathrm{abc}}+P_{S}\left[t_{i}^{a}\left(\mathrm{jb}|\mathrm{kc}\right)\right]$$ 19

*P_L_* and *P_S_*, or the “long” and “short” permutation operators, are defined as

20 $$P_{L}\left(A_{\mathrm{ijk}}^{\mathrm{abc}}\right)=A_{\mathrm{ijk}}^{\mathrm{abc}}+A_{\mathrm{ikj}}^{\mathrm{acb}}+A_{\mathrm{jik}}^{\mathrm{bac}}+A_{\mathrm{jki}}^{\mathrm{bca}}+A_{\mathrm{kij}}^{\mathrm{cab}}+A_{\mathrm{kji}}^{\mathrm{cba}}$$

21 $$P_{S}\left(A_{\mathrm{ijk}}^{\mathrm{abc}}\right)=A_{\mathrm{ijk}}^{\mathrm{abc}}+A_{\mathrm{jik}}^{\mathrm{bac}}+A_{\mathrm{kij}}^{\mathrm{cab}}$$

If the λ_1_ and λ_2_ expressions are used, then the expression becomes, using an analogously defined intermediate

$$\Lambda_{\mathrm{ijk}}^{\mathrm{abc}}=P_{L}\left[\left(\mathrm{ia}|\mathrm{bd}\right)\lambda_{\mathrm{kj}}^{\mathrm{cd}}-\left(\mathrm{ia}|\mathrm{jl}\right)\lambda_{\mathrm{kl}}^{\mathrm{cb}}\right]+P_{S}\left[\lambda_{i}^{a}\left(\mathrm{jb}|\mathrm{kc}\right)\right]$$ 22

Thus, the analogous (*T*)_Λ_ energy expression is

23 $$E_{{\left(T\right)}_{\Lambda }}=T_{\mathrm{ijk}}^{\mathrm{abc}}\cdot \left(\frac{4}{3}\Lambda_{\mathrm{ijk}}^{\mathrm{abc}}-2\Lambda_{\mathrm{ijk}}^{\mathrm{cba}}+\frac{2}{3}\Lambda_{\mathrm{ijk}}^{\mathrm{cab}}\right)$$

This expression is also known as the “asymmetric” triples correction, since λ-like quantities are used on one side, and *T*-like quantities were used on the other.

### IV.C Overview of Domain-Based Local Pair Natural Orbital (DLPNO) Approach

The domain-based pair natural orbital (DLPNO) approach, initially developed from the work of Neese and co-workers,^69–72^ and extended to higher levels of coupled-cluster theory in our prior work,^83–85^ involves assigning domain-specific occupied, virtual, as well as auxiliary function domains for density-fitted integrals. Domains can be single occupied orbitals *i*, pairs of occupied orbitals *ij*, triplets *ijk*, as well as quadruplets *ijkl*. For a more comprehensive overview, check out our prior work.^85^

#### IV.C.I Local Occupied Domain

First, the active occupied orbitals from the completion of a preceding Hartree–Fock computation are localized through a unitary transformation

24 $$C_{\mu i}^{L}=\sum_{j\in \mathrm{active}\;\mathrm{occ}}C_{\mu j}U_{\mathrm{ji}}$$

where *U* is defined by optimizing a particular metric over the transformed orbitals, such as minimizing spatial extent in the Foster-Boys approach,^87,88^ maximizing partial charge per center in the Pipek-Mezey approach,^88,89^ or maximizing orbital self-repulsion in the Edmiston-Ruedenberg approach.^90,91^ These transformed occupied orbitals are known as local molecular orbitals (LMOs).

Once these orbitals are localized, various prescreening metrics are performed on every possible pair of occupied orbitals *ij* based on sparsity metrics, such that after all the prescreening is complete, there are only O(N) interacting pairs as a function of system size. Once these significant pairs are determined, interacting triplets, quadruplets, etc. are determined from these pairs. A triplet *ijk* is only considered if *ij*, *jk*, and *ik* are significant pairs, and a quadruplet *ijkl* is also only considered if *ij*, *ik*, *il*, *jk*, *jl*, and *kl* are all significant pairs. These domains can also be further truncated, and their energies accounted for with a corresponding perturbation theory. At the DLPNO–CCSD level of theory, pairs are further prescreened using semicanonical local MP2;^77,82^ at DLPNO–CCSD­(T)/T, triplets are prescreened using semicanonical DLPNO–CCSD­(T_0_),^82,83^ and at the DLPNO–CCSDT­(*Q*)/*Q* level, quadruplets are screened using semicanonical DLPNO–CCSDT­(*Q*_0_).^84,85^ Once these domains are determined, local occupied domains can be determined. For each occupied orbital *i*, a local occupied domain *m*_*i*_ is defined as the set of all *m* such that *im* is a significant pair. For every pair of occupied orbitals *ij*, the local occupied domain *m*_*ij*_ is defined as the set of all *m* such that *im* and *jm* are valid pairs. For every triplet *ijk*, *m*_*ijk*_ is defined such that *im*, *jm*, and *km* form valid pairs; and for quadruplet *ijkl*, *m*_*ijkl*_ is correspondingly formed from the intersection of *im*, *jm*, *km*, and *lm*.

#### IV.C.II Local Virtual Domains

Local virtual domains are initially assigned to each domain through projected atomic orbitals (PAOs).^54^ Projected atomic orbitals form a sparse, but linearly dependent representation of the virtual space through the subtraction of the span of the occupied space from the complete AO space

25 $$C_{\mu \mathrm{\nu ̃}}^{\mathrm{PAO}}=\delta_{\mu \nu }-C_{\mu i}^{\mathrm{MO}}C_{\sigma i}^{\mathrm{MO}}S_{\sigma \nu }^{\mathrm{AO}}$$

26 $$S_{\mathrm{\mu ̃}\mathrm{\nu ̃}}^{\mathrm{PAO}}=C_{\lambda \mathrm{\mu ̃}}^{\mathrm{PAO}}S_{\lambda \sigma }^{\mathrm{AO}}C_{\sigma \mathrm{\nu ̃}}^{\mathrm{PAO}}$$

27 $$C_{\mu \mathrm{\nu ̃}}^{\mathrm{PAO}}\leftarrow {\left(S_{\mathrm{\nu ̃}\mathrm{\nu ̃}}^{\mathrm{PAO}}\right)}^{-1/2}C_{\mu \mathrm{\nu ̃}}^{\mathrm{PAO}}$$

After the PAOs are computed, PAO domains μ̃_*i*_ are formed from the set of all PAOs μ̃ that have significant overlap with LMO *i*. This is done through sparsity measures such as the “differential overlap integral”

28 $${\mathrm{DOI}}_{i\mathrm{\mu ̃}}={\left(\int \left|\phi_{i}\left(r\right){|}^{2}\right|\phi_{\mathrm{\mu ̃}}\left(r\right){|}^{2}dr\right)}^{1/2}$$

The corresponding PAO domains μ̃_*ij*_ are formed from the union of domains μ̃_*i*_ and μ̃_*j*_; μ̃_*ijk*_ from the union of μ̃_*i*_, μ̃_*j*_, and μ̃_*k*_; μ̃_*ijkl*_ from μ̃_*i*_, μ̃_*j*_, μ̃_*k*_, and μ̃_*l*_. Once these PAO domains are determined, localized natural orbital spaces can be formed from the diagonalization of a corresponding domain-based density. For a pair *ij*, the pair density is defined, using MP2 amplitudes *t*_*ij*_^*ab*^, with antisymmetrized amplitudes *u*_*ij*_^*ab*^ = 2*t*_*ij*_^*ab*^ – *t*_*ij*_^*ba*^

29 $$D_{\mathrm{ij}}^{\mathrm{ab}}=\frac{1}{1+\delta_{\mathrm{ij}}}\left[u_{\mathrm{ij}}^{\mathrm{ac}}t_{\mathrm{ij}}^{\mathrm{bc}}+u_{\mathrm{ij}}^{\mathrm{ca}}t_{\mathrm{ij}}^{\mathrm{cb}}\right]$$

These amplitudes are formed in the basis of the corresponding PAO domains μ̃_*ij*_, but canonicalized to remove linear-dependencies. Once the pair density is diagonalized, a truncated basis can be formed by truncating the orbitals by eigenvalue, also known as occupation numbers. After this new truncated basis is canonicalized, the resulting basis is known as the pair natural orbitals (PNOs). These are denoted *a*_*ij*_, *b*_*ij*_ for pairs. Single occupied orbitals *i* are assigned diagonal PNOs *a*_*ii*_, *b*_*ii*_. For triplets *ijk*, the triplet density $D_{\mathrm{ijk}}=\frac{1}{3}\left(D_{\mathrm{ij}}+D_{\mathrm{jk}}+D_{\mathrm{ik}}\right)$ is diagonalized, computed from converged CCSD doubles amplitudes projected to canonicalized μ̃_*ijk*_. The resulting orbitals are known as triples natural orbitals (TNOs). For quadruplets *ijkl*, the quadruples density is formed from $D_{\mathrm{ijk}}=\frac{1}{6}\left(D_{\mathrm{ij}}+D_{\mathrm{ik}}+D_{\mathrm{il}}+D_{\mathrm{jk}}+D_{\mathrm{jl}}+D_{\mathrm{kl}}\right)$ , using converged CCSDT doubles amplitudes canonicalized μ̃_*ijkl*_, forming quadruples natural orbitals (QNOs). This “cascaded” approach of initially assigning PAOs to each domain before localized natural orbitals are formed is an innovation of Frank Neese and co-workers in their original DLPNO–CCSD method,^71^ and is now commonly used in local pair natural orbital based methods.

#### IV.C.III Local Auxiliary/Fitting Domain

To reduce the cost of forming two-electron integrals, the density-fitting (DF)/resolution of the identity (RI) approximation is used, involving the use of atom-centered auxiliary basis functions *P*, *Q*.^92–100^

30 $$\left(\mathrm{pq}|\mathrm{rs}\right)\approx B_{\mathrm{pq}}^{Q}B_{\mathrm{rs}}^{Q}$$

where

31 $$B_{\mathrm{pq}}^{Q}={\left(Q|P\right)}^{-1/2}\left(P|\mathrm{pq}\right)$$

To ensure that the formation of the integrals remains linear-scaling, though without loss of accuracy, a localized auxiliary domain *P*_*i*_ for each LMO *i* is determined from the union of all atoms such that the Mulliken population of electrons from LMO *i* exceeds a given tolerance. The Mulliken population of electrons on LMO *i* on an atomic center *A* is computed through:

32 $$P_{\mu \nu }^{i}=C_{\mu i}^{L}S_{\mu \nu }C_{\nu i}^{L}$$

33 $$q_{\mathrm{iA}}=2\sum_{\mu \in A}\sum_{\nu }P_{\mu \nu }^{i}\cdot \frac{P_{\mu \mu }^{i}}{P_{\mu \mu }^{i}+P_{\nu \nu }^{i}}$$

After each domain *P*_*i*_, pair domains *P*_*ij*_ can be formed from the union of *P*_*i*_ and *P*_*j*_; triplets *P*_*ijk*_ from *P*_*i*_, *P*_*j*_, and *P*_*k*_; and quadruplets *P*_*ijkl*_ from *P*_*i*_, *P*_*j*_, *P*_*k*_, and *P*_*l*_.

### IV.D Derivation of DLPNO–CCSD Λ Equations

We derive our working equations in the spirit of our prior research,^82^ where the equations for *l*_*i*_^*a*_*ii*_^ and *l*_*ij*_^*a*_*ij*_ *b*_*ij*_^ are derived from taking the derivative of the PNO Lagrangian contracted with the residual equations as presented in that work. The equations would look different from the DLPNO–CCSD Lambda equations as available in the ORCA package,^101^ derived by Datta et al.^86^ since our DLPNO–CCSD working equations^82^ are different than the original formulation by Riplinger, Neese, and co-workers.^71^

34 $$L=E_{\mathrm{CCSD}}+\sum_{m,e_{\mathrm{mm}}}\lambda_{m}^{e_{\mathrm{mm}}}R_{m}^{e_{\mathrm{mm}}}+\sum_{m,n,e_{\mathrm{mn}},f_{\mathrm{mn}}}\lambda_{\mathrm{mn}}^{e_{\mathrm{mn}}f_{\mathrm{mn}}}R_{\mathrm{mn}}^{e_{\mathrm{mn}}f_{\mathrm{mn}}}$$

35 $$l_{i}^{a_{\mathrm{ii}}}=\frac{\partial L}{\partial T_{i}^{a_{\mathrm{ii}}}}=2S_{a_{\mathrm{in}}}^{a_{\mathrm{ii}}}L_{\mathrm{in}}^{a_{\mathrm{in}}f_{\mathrm{in}}}S_{f_{\mathrm{nn}}}^{f_{\mathrm{in}}}T_{n}^{f_{\mathrm{nn}}}+\sum_{m,e_{\mathrm{mm}}}\lambda_{m}^{e_{\mathrm{mm}}}\frac{\partial R_{m}^{e_{\mathrm{mm}}}}{\partial T_{i}^{a_{\mathrm{ii}}}}+\sum_{m,n,e_{\mathrm{mn}},f_{\mathrm{mn}}}\lambda_{\mathrm{mn}}^{e_{\mathrm{mn}}f_{\mathrm{mn}}}\frac{\partial R_{\mathrm{mn}}^{e_{\mathrm{mn}}f_{\mathrm{mn}}}}{\partial T_{i}^{a_{\mathrm{ii}}}}$$

36 $$l_{\mathrm{ij}}^{a_{\mathrm{ij}}b_{\mathrm{ij}}}=\frac{\partial L}{\partial T_{\mathrm{ij}}^{a_{\mathrm{ij}}b_{\mathrm{ij}}}}=L_{\mathrm{ij}}^{a_{\mathrm{ij}}b_{\mathrm{ij}}}+\sum_{m,e_{\mathrm{mm}}}\lambda_{m}^{e_{\mathrm{mm}}}\frac{\partial R_{m}^{e_{\mathrm{mm}}}}{\partial T_{\mathrm{ij}}^{a_{\mathrm{ij}}b_{\mathrm{ij}}}}+\sum_{m,n,e_{\mathrm{mn}},f_{\mathrm{mn}}}\lambda_{\mathrm{mn}}^{e_{\mathrm{mn}}f_{\mathrm{mn}}}\frac{\partial R_{\mathrm{mn}}^{e_{\mathrm{mn}}f_{\mathrm{mn}}}}{\partial T_{\mathrm{ij}}^{a_{\mathrm{ij}}b_{\mathrm{ij}}}}$$

where the first term of eq 35 comes from taking the derivative $\frac{\partial E_{\mathrm{CCSD}}}{\partial T_{i}^{a_{\mathrm{ii}}}}$ and the first term of eq 36 comes from taking the derivative $\frac{\partial E_{\mathrm{CCSD}}}{\partial T_{\mathrm{ij}}^{a_{\mathrm{ij}}b_{\mathrm{ij}}}}$ . We introduce the following notation for expressing integrals in our *t*_1_-dressed lambda equations, for integrals and Fock matrix elements, using the same convention as in prior work^82^

37 $$J_{\mathrm{pq}}^{\mathrm{rs}}=\left(\mathrm{pq}|\mathrm{rs}\right)=B_{\mathrm{pq}}^{Q}B_{\mathrm{rs}}^{Q}$$

38 $$K_{\mathrm{pq}}^{\mathrm{rs}}=\left(\mathrm{pr}|\mathrm{qs}\right)=B_{\mathrm{pr}}^{Q}B_{\mathrm{qs}}^{Q}$$

39 $$L_{\mathrm{pq}}^{\mathrm{rs}}=2K_{\mathrm{pq}}^{\mathrm{rs}}-K_{\mathrm{pq}}^{\mathrm{sr}}$$

40 $$M_{\mathrm{pq}}^{\mathrm{rs}}=2K_{\mathrm{pq}}^{\mathrm{rs}}-J_{\mathrm{pq}}^{\mathrm{rs}}$$

41 $$N_{\mathrm{pq}}^{\mathrm{rs}}=2J_{\mathrm{pq}}^{\mathrm{rs}}-K_{\mathrm{pq}}^{\mathrm{rs}}$$

The *t*_1_-dressed form of density-fitted ERIs and Fock matrix elements take the form, with *T̃*_*m*_^*e*_*kl*_^ = *T*_*m*_^*e*_*mm*_^ *S*_*e*_*mm*__^*e*_*kl*_^ representing PNO-projected *T*_1_ amplitudes

42 $${\mathrm{B̃}}_{\mathrm{ki}}^{Q_{\mathrm{ij}}}=B_{\mathrm{ki}}^{Q_{\mathrm{ij}}}+B_{ka_{\mathrm{ij}}}^{Q_{\mathrm{ij}}}{\mathrm{T̃}}_{i}^{a_{\mathrm{ij}}}$$

43 $${\mathrm{B̃}}_{a_{\mathrm{ij}}i}^{Q_{\mathrm{ij}}}=B_{a_{\mathrm{ij}}i}^{Q_{\mathrm{ij}}}-{\mathrm{T̃}}_{k}^{a_{\mathrm{ij}}}B_{\mathrm{ki}}^{Q_{\mathrm{ij}}}+B_{a_{\mathrm{ij}}b_{\mathrm{ij}}}^{Q_{\mathrm{ij}}}{\mathrm{T̃}}_{i}^{b_{\mathrm{ij}}}-{\mathrm{T̃}}_{k}^{a_{\mathrm{ij}}}B_{kb_{\mathrm{ij}}}^{Q_{\mathrm{ij}}}{\mathrm{T̃}}_{i}^{b_{\mathrm{ij}}}$$

44 $${\mathrm{B̃}}_{a_{\mathrm{ij}}b_{\mathrm{ij}}}^{Q_{\mathrm{ij}}}=B_{a_{\mathrm{ij}}b_{\mathrm{ij}}}^{Q_{\mathrm{ij}}}-{\mathrm{T̃}}_{k}^{a_{\mathrm{ij}}}B_{kb_{\mathrm{ij}}}^{Q_{\mathrm{ij}}}$$

45 $${\mathrm{F̃}}_{\mathrm{ij}}={\mathrm{F̅}}_{\mathrm{ij}}+{\mathrm{F̅}}_{ic_{\mathrm{jj}}}T_{j}^{c_{\mathrm{jj}}}$$

46 $${\mathrm{F̃}}_{ia_{\mathrm{ij}}}=S_{a_{\mathrm{ik}}}^{a_{\mathrm{ij}}}L_{\mathrm{ik}}^{a_{\mathrm{ik}}c_{\mathrm{ik}}}{\mathrm{T̃}}_{k}^{c_{\mathrm{ik}}}$$

47 $${\mathrm{F̃}}_{a_{\mathrm{ii}}i}={\mathrm{F̅}}_{a_{\mathrm{ii}}i}-{\mathrm{T̃}}_{k}^{a_{\mathrm{ii}}}{\mathrm{F̅}}_{\mathrm{ki}}+{\mathrm{F̅}}_{a_{\mathrm{ii}}b_{\mathrm{ii}}}T_{i}^{b_{\mathrm{ii}}}-{\mathrm{T̃}}_{k}^{a_{\mathrm{ii}}}{\mathrm{F̅}}_{kb_{\mathrm{ii}}}T_{i}^{b_{\mathrm{ii}}}$$

48 $${\mathrm{F̃}}_{a_{\mathrm{ij}}b_{\mathrm{ij}}}={\mathrm{F̅}}_{a_{\mathrm{ij}}b_{\mathrm{ij}}}-{\mathrm{T̃}}_{k}^{a_{\mathrm{ij}}}{\mathrm{F̅}}_{kb_{\mathrm{ij}}}$$

where

49 $${\mathrm{F̅}}_{\mathrm{ij}}=F_{\mathrm{ij}}+\left[2J_{\mathrm{ij}}^{kc_{\mathrm{ij}}}-K_{\mathrm{ji}}^{kc_{\mathrm{ij}}}\right]{\mathrm{T̃}}_{k}^{c_{\mathrm{ij}}}$$

50 $${\mathrm{F̅}}_{ia_{\mathrm{kl}}}=\left[2B_{ia_{\mathrm{kl}}}^{Q_{\mathrm{kl}}}B_{mc_{\mathrm{kl}}}^{Q_{\mathrm{kl}}}-B_{ic_{\mathrm{kl}}}^{Q_{\mathrm{kl}}}B_{ma_{\mathrm{kl}}}^{Q_{\mathrm{kl}}}\right]{\mathrm{T̃}}_{m}^{c_{\mathrm{kl}}}$$

51 $${\mathrm{F̅}}_{a_{\mathrm{ii}}i}=\left[2B_{a_{\mathrm{ii}}i}^{Q_{\mathrm{ii}}}B_{kc_{\mathrm{ii}}}^{Q_{\mathrm{ii}}}-B_{a_{\mathrm{ii}}c_{\mathrm{ii}}}^{Q_{\mathrm{ii}}}B_{\mathrm{ki}}^{Q_{\mathrm{ii}}}\right]{\mathrm{T̃}}_{k}^{c_{\mathrm{ii}}}$$

52 $${\mathrm{F̅}}_{a_{\mathrm{ij}}b_{\mathrm{ij}}}=\epsilon_{a_{\mathrm{ij}}}\delta_{a_{\mathrm{ij}}b_{\mathrm{ij}}}+\left[2B_{a_{\mathrm{ij}}b_{\mathrm{ij}}}^{Q_{\mathrm{ij}}}B_{kc_{\mathrm{ij}}}^{Q_{\mathrm{ij}}}-B_{a_{\mathrm{ij}}c_{\mathrm{ij}}}^{Q_{\mathrm{ij}}}B_{kb_{\mathrm{ij}}}^{Q_{\mathrm{ij}}}\right]{\mathrm{T̃}}_{k}^{c_{\mathrm{ij}}}$$

The density-like ρ intermediates are defined in the PNO basis as

53 $$\rho_{\mathrm{nk}}^{\mathrm{OO}}=\sum_{m}\lambda_{\mathrm{mn}}^{e_{\mathrm{mn}}f_{\mathrm{mn}}}\left(\sum_{e_{\mathrm{mk}},f_{\mathrm{mk}}}S_{e_{\mathrm{mk}}}^{e_{\mathrm{mn}}}T_{\mathrm{mk}}^{e_{\mathrm{mk}}f_{\mathrm{mk}}}S_{f_{\mathrm{mk}}}^{f_{\mathrm{mn}}}\right)$$

54 $$\rho_{f_{\mathrm{mn}}c_{\mathrm{mn}}}^{\mathrm{VV}}=\sum_{e_{\mathrm{mn}}}\lambda_{\mathrm{mn}}^{e_{\mathrm{mn}}f_{\mathrm{mn}}}T_{\mathrm{mn}}^{e_{\mathrm{mn}}c_{\mathrm{mn}}}$$

In addition, we present another intermediate used extensively in our derivations

55 $${\mathrm{\lambda ̅}}_{\mathrm{mn}}^{e_{\mathrm{mn}}f_{\mathrm{mn}}}=\frac{1}{2}\lambda_{\mathrm{mn}}^{e_{\mathrm{mn}}f_{\mathrm{mn}}}+\lambda_{\mathrm{mn}}^{f_{\mathrm{mn}}e_{\mathrm{mn}}}$$

#### IV.D.I *l*_*i*_^*a*_*ii*_^ Residual

The first contribution to *l*_*i*_^*a*_*ii*_^ arises from $\frac{\partial E_{\mathrm{CCSD}}}{\partial T_{i}^{a_{\mathrm{ii}}}}$

$$l_{i}^{a_{\mathrm{ii}}}+=2\left(S_{a_{\mathrm{in}}}^{a_{\mathrm{ii}}}L_{\mathrm{in}}^{a_{\mathrm{in}}f_{\mathrm{in}}}S_{f_{\mathrm{nn}}}^{f_{\mathrm{in}}}\right)T_{n}^{f_{\mathrm{nn}}}$$

Next, we present the λ_1_ contributions to *l*_*i*_^*a*_*ii*_^, the terms that arise from λ_*m*_^*e*_*mm*_^∂*R*_*m*_^*e*_*mm*_^/∂*T*_*i*_^*a*_*ii*_^. First we define these intermediates, which only need to be computed once, prior to the Lambda CCSD iterations

56 $${\mathrm{M̃}}_{\mathrm{im}}^{a_{\mathrm{ii}}e_{\mathrm{mm}}}=S_{a_{\mathrm{mm}}}^{a_{\mathrm{ii}}}M_{\mathrm{im}}^{a_{\mathrm{mm}}e_{\mathrm{mm}}}-{\mathrm{T̃}}_{k}^{e_{\mathrm{mm}}}N_{\mathrm{mk}}^{ia_{\mathrm{mk}}}S_{a_{\mathrm{mk}}}^{a_{\mathrm{ii}}}+M_{if_{\mathrm{mm}}}^{a_{\mathrm{mm}}e_{\mathrm{mm}}}S_{a_{\mathrm{ii}}}^{a_{\mathrm{mm}}}T_{m}^{f_{\mathrm{mm}}}-{\mathrm{T̃}}_{k}^{e_{\mathrm{mm}}}L_{\mathrm{ik}}^{a_{\mathrm{mm}}f_{\mathrm{mm}}}S_{a_{\mathrm{ii}}}^{a_{\mathrm{mm}}}T_{m}^{f_{\mathrm{mm}}}$$

57 $${\mathrm{\delta ̃}}_{\mathrm{im}}^{a_{\mathrm{ii}}e_{\mathrm{mm}}}={\mathrm{M̃}}_{\mathrm{im}}^{a_{\mathrm{ii}}e_{\mathrm{mm}}}+S_{a_{\mathrm{ik}}}^{a_{\mathrm{ii}}}L_{\mathrm{ik}}^{a_{\mathrm{ik}}c_{\mathrm{ik}}}S_{c_{\mathrm{km}}}^{c_{\mathrm{ik}}}U_{\mathrm{km}}^{c_{\mathrm{km}}e_{\mathrm{km}}}S_{e_{\mathrm{mm}}}^{e_{\mathrm{km}}}$$

The λ_1_ contributions to *l*_*i*_^*a*_*ii*_^ take the form

58 $$l_{i}^{a_{\mathrm{ii}}}+={\mathrm{\delta ̃}}_{\mathrm{im}}^{a_{\mathrm{ii}}e_{\mathrm{mm}}}\lambda_{m}^{e_{\mathrm{mm}}}-{\tilde{\mathrm{F̃}}}_{\mathrm{im}}\lambda_{m}^{a_{\mathrm{mm}}}S_{a_{\mathrm{mm}}}^{a_{\mathrm{ii}}}+\lambda_{i}^{e_{\mathrm{ii}}}{\tilde{\mathrm{F̃}}}_{e_{\mathrm{ii}}a_{\mathrm{ii}}}$$

Next, we present the λ_2_ contributions to *l*_*i*_^*a*_*ii*_^, the terms that arise from λ_*mn*_^*e*_*mn*_ *f*_*mn*_^∂*R*_*mn*_^*e*_*mn*_ *f*_*mn*_^/∂*T*_*i*_^*a*_*ii*_^. With the noniterative intermediates

59 $${\tilde{\mathrm{K̃}}}_{ma_{\mathrm{ii}}}^{e_{\mathrm{mi}}f_{\mathrm{mi}}}=\left[{\mathrm{B̃}}_{e_{\mathrm{mi}}m}^{Q_{\mathrm{mi}}}{\mathrm{B̃}}_{f_{\mathrm{mi}}a_{\mathrm{mi}}}^{Q_{\mathrm{mi}}}+\left(S_{e_{\mathrm{kl}}}^{e_{\mathrm{mi}}}T_{\mathrm{kl}}^{e_{\mathrm{kl}}f_{\mathrm{kl}}}S_{f_{\mathrm{kl}}}^{f_{\mathrm{mi}}}\right){\mathrm{B̃}}_{\mathrm{km}}^{Q_{\mathrm{mi}}}B_{la_{\mathrm{mi}}}^{Q_{\mathrm{mi}}}\right]S_{a_{\mathrm{mi}}}^{a_{\mathrm{ii}}}-\left(S_{e_{\mathrm{mk}}}^{e_{\mathrm{mi}}}T_{\mathrm{mk}}^{e_{\mathrm{mk}}f_{\mathrm{mk}}}S_{f_{\mathrm{mk}}}^{f_{\mathrm{mi}}}\right){\mathrm{F̅}}_{ka_{\mathrm{ii}}}$$

60 $${\tilde{\mathrm{K̃}}}_{e_{\mathrm{mn}}i}^{\mathrm{mn}}=\left[{\mathrm{B̃}}_{e_{\mathrm{mn}}m}^{Q_{\mathrm{mn}}}{\mathrm{B̃}}_{\mathrm{in}}^{Q_{\mathrm{mn}}}+T_{\mathrm{mn}}^{c_{\mathrm{mn}}d_{\mathrm{mn}}}{\mathrm{B̃}}_{e_{\mathrm{mn}}c_{\mathrm{mn}}}^{Q_{\mathrm{mn}}}B_{id_{\mathrm{mn}}}^{Q_{\mathrm{mn}}}\right]+T_{\mathrm{mn}}^{e_{\mathrm{mn}}c_{\mathrm{mn}}}{\mathrm{F̅}}_{ic_{\mathrm{mn}}}$$

61 $${\mathrm{M̅}}_{ka_{\mathrm{ii}}}^{c_{\mathrm{ki}}e_{\mathrm{ki}}}=S_{a_{\mathrm{ki}}}^{a_{\mathrm{ii}}}M_{ka_{\mathrm{ki}}}^{c_{\mathrm{ki}}e_{\mathrm{ki}}}-\left(S_{a_{\mathrm{lk}}}^{a_{\mathrm{ii}}}L_{\mathrm{lk}}^{a_{\mathrm{lk}}c_{\mathrm{lk}}}S_{c_{\mathrm{lk}}}^{c_{\mathrm{ki}}}\right){\mathrm{T̃}}_{l}^{e_{\mathrm{ki}}}$$

62 $${\mathrm{M̅}}_{\mathrm{mk}}^{ic_{\mathrm{mk}}}=M_{\mathrm{mk}}^{ic_{\mathrm{mk}}}+{\mathrm{T̃}}_{m}^{f_{\mathrm{ik}}}L_{\mathrm{ik}}^{f_{\mathrm{ik}}c_{\mathrm{ik}}}S_{c_{\mathrm{ik}}}^{c_{\mathrm{km}}}$$

63 $${\mathrm{J̅}}_{\mathrm{km}}^{ic_{\mathrm{km}}}=J_{\mathrm{km}}^{ic_{\mathrm{km}}}+{\mathrm{T̃}}_{m}^{f_{\mathrm{ki}}}K_{\mathrm{ki}}^{f_{\mathrm{ki}}c_{\mathrm{ki}}}S_{c_{\mathrm{ki}}}^{c_{\mathrm{km}}}$$

64 $${\mathrm{J̅}}_{ka_{\mathrm{ii}}}^{e_{\mathrm{ki}}c_{\mathrm{ki}}}=\left[J_{ka_{\mathrm{ki}}}^{e_{\mathrm{ki}}c_{\mathrm{ki}}}S_{a_{\mathrm{ki}}}^{a_{\mathrm{ii}}}-S_{a_{\mathrm{kl}}}^{a_{\mathrm{ii}}}K_{\mathrm{kl}}^{a_{\mathrm{kl}}c_{\mathrm{kl}}}S_{c_{\mathrm{kl}}}^{c_{\mathrm{ki}}}{\mathrm{T̃}}_{l}^{e_{\mathrm{ki}}}\right]$$

65 $${\mathrm{F̂}}_{f_{\mathrm{mn}}c_{\mathrm{mn}}}^{ia_{\mathrm{ii}}}=\left[2{\mathrm{B̃}}_{f_{\mathrm{mn}}c_{\mathrm{mn}}}^{Q_{\mathrm{mn}}}B_{ia_{\mathrm{mn}}}^{Q_{\mathrm{mn}}}-{\mathrm{B̃}}_{f_{\mathrm{mn}}a_{\mathrm{mn}}}^{Q_{\mathrm{mn}}}B_{ic_{\mathrm{mn}}}^{Q_{\mathrm{mn}}}\right]S_{a_{\mathrm{mn}}}^{a_{\mathrm{ii}}}$$

66 $${\mathrm{F̂}}_{\mathrm{kn}}^{ia_{\mathrm{ii}}}=\left[2J_{\mathrm{kn}}^{ia_{\mathrm{kn}}}-K_{\mathrm{nk}}^{ia_{\mathrm{kn}}}\right]S_{a_{\mathrm{ii}}}^{a_{\mathrm{kn}}}+S_{a_{\mathrm{ii}}}^{a_{\mathrm{nn}}}\left[2B_{kc_{\mathrm{nn}}}^{Q_{\mathrm{nn}}}B_{ia_{\mathrm{nn}}}^{Q_{\mathrm{nn}}}-B_{ka_{\mathrm{nn}}}^{Q_{\mathrm{nn}}}B_{ic_{\mathrm{nn}}}^{Q_{\mathrm{nn}}}\right]T_{n}^{c_{\mathrm{nn}}}$$

The λ_2_ contributions to *l*_*i*_^*a*_*ii*_^ take the form

67 $$l_{i}^{a_{\mathrm{ii}}}+={\tilde{\mathrm{K̃}}}_{ma_{\mathrm{ii}}}^{e_{\mathrm{mi}}f_{\mathrm{mi}}}\lambda_{\mathrm{mi}}^{e_{\mathrm{mi}}f_{\mathrm{mi}}}-{\tilde{\mathrm{K̃}}}_{e_{\mathrm{mn}}i}^{\mathrm{mn}}\lambda_{\mathrm{mn}}^{e_{\mathrm{mn}}a_{\mathrm{mn}}}S_{a_{\mathrm{mn}}}^{a_{\mathrm{ii}}}$$

68 $$l_{i}^{a_{\mathrm{ii}}}+=\rho_{f_{\mathrm{mn}}c_{\mathrm{mn}}}^{\mathrm{VV}}{\mathrm{F̂}}_{f_{\mathrm{mn}}c_{\mathrm{mn}}}^{ia_{\mathrm{ii}}}-\rho_{\mathrm{nk}}^{\mathrm{OO}}{\mathrm{F̂}}_{\mathrm{kn}}^{ia_{\mathrm{ii}}}$$

69 $$l_{i}^{a_{\mathrm{ii}}}+=S_{a_{\mathrm{km}}}^{a_{\mathrm{ii}}}\left(S_{a_{\mathrm{mn}}}^{a_{\mathrm{km}}}{\mathrm{\lambda ̅}}_{\mathrm{mn}}^{a_{\mathrm{mn}}f_{\mathrm{mn}}}S_{f_{\mathrm{mn}}}^{f_{\mathrm{kn}}}\right)T_{\mathrm{kn}}^{f_{\mathrm{kn}}c_{\mathrm{kn}}}S_{c_{\mathrm{km}}}^{c_{\mathrm{kn}}}{\mathrm{J̅}}_{\mathrm{km}}^{ic_{\mathrm{km}}}$$

70 $$l_{i}^{a_{\mathrm{ii}}}-=\left(S_{e_{\mathrm{in}}}^{e_{\mathrm{ki}}}{\mathrm{\lambda ̅}}_{\mathrm{in}}^{e_{\mathrm{in}}f_{\mathrm{in}}}S_{f_{\mathrm{kn}}}^{f_{\mathrm{in}}}\right)T_{\mathrm{kn}}^{f_{\mathrm{kn}}c_{\mathrm{kn}}}S_{c_{\mathrm{ki}}}^{c_{\mathrm{kn}}}{\mathrm{J̅}}_{ka_{\mathrm{ii}}}^{e_{\mathrm{ki}}c_{\mathrm{ki}}}$$

71 $$l_{i}^{a_{\mathrm{ii}}}+=\frac{1}{2}S_{e_{\mathrm{in}}}^{e_{\mathrm{ik}}}\lambda_{\mathrm{in}}^{e_{\mathrm{in}}f_{\mathrm{in}}}\left(S_{f_{\mathrm{nk}}}^{f_{\mathrm{in}}}U_{\mathrm{nk}}^{f_{\mathrm{nk}}c_{\mathrm{nk}}}S_{c_{\mathrm{ki}}}^{c_{\mathrm{nk}}}\right){\mathrm{M̅}}_{ka_{\mathrm{ii}}}^{c_{\mathrm{ki}}e_{\mathrm{ki}}}$$

72 $$l_{i}^{a_{\mathrm{ii}}}-=\frac{1}{2}S_{a_{\mathrm{mk}}}^{a_{\mathrm{ii}}}\left(S_{a_{\mathrm{mn}}}^{a_{\mathrm{mk}}}\lambda_{\mathrm{mn}}^{a_{\mathrm{mn}}f_{\mathrm{mn}}}S_{f_{\mathrm{nk}}}^{f_{\mathrm{mn}}}\right)U_{\mathrm{nk}}^{f_{\mathrm{nk}}c_{\mathrm{nk}}}S_{c_{\mathrm{mk}}}^{c_{\mathrm{nk}}}{\mathrm{M̅}}_{\mathrm{mk}}^{ic_{\mathrm{mk}}}$$

#### IV.D.II *l*_*ij*_^*a*_*ij*_ *b*_*ij*_^ Residual

The contribution to *l*_*ij*_^*a*_*ij*_ *b*_*ij*_^ from $\frac{\partial E_{\mathrm{CCSD}}}{\partial T_{\mathrm{ij}}^{a_{\mathrm{ij}}b_{\mathrm{ij}}}}$ takes the form, with *l*_*ij*_^*ab*^ = *l̅*_*ij*_^*ab*^ + *l̅*_*ji*_^*ba*^

$${\mathrm{l̅}}_{\mathrm{ij}}^{a_{\mathrm{ij}}b_{\mathrm{ij}}}+=L_{\mathrm{ij}}^{a_{\mathrm{ij}}b_{\mathrm{ij}}}$$

Next, we present the λ_1_ contributions to *l*_*ij*_^*a*_*ij*_ *b*_*ij*_^, the terms that arise from λ_*m*_^*e*_*mm*_^∂*R*_*m*_^*e*_*mm*_^/∂*T*_*ij*_^*a*_*ij*_ *b*_*ij*_^. First, we present the noniterative intermediates

73 $${\mathrm{L̅}}_{ie_{\mathrm{jj}}}^{a_{\mathrm{ij}}b_{\mathrm{ij}}}=L_{ie_{\mathrm{ij}}}^{a_{\mathrm{ij}}b_{\mathrm{ij}}}S_{e_{\mathrm{ij}}}^{e_{\mathrm{jj}}}-{\mathrm{T̃}}_{l}^{e_{\mathrm{jj}}}\left[S_{a_{\mathrm{il}}}^{a_{\mathrm{ij}}}L_{\mathrm{il}}^{a_{\mathrm{il}}b_{\mathrm{il}}}S_{b_{\mathrm{il}}}^{b_{\mathrm{ij}}}\right]$$

74 $${\mathrm{K̅}}_{\mathrm{ij}}^{mb_{\mathrm{ij}}}=K_{\mathrm{ij}}^{mb_{\mathrm{ij}}}+{\mathrm{T̃}}_{m}^{e_{\mathrm{ij}}}K_{\mathrm{ij}}^{e_{\mathrm{ij}}b_{\mathrm{ij}}}$$

Hence, the λ_1_ contributions to *l*_*ij*_^*a*_*ij*_ *b*_*ij*_^ takes the form

75 $$l_{\mathrm{ij}}^{a_{\mathrm{ij}}b_{\mathrm{ij}}}+=\lambda_{j}^{e_{\mathrm{jj}}}{\mathrm{L̅}}_{ie_{\mathrm{jj}}}^{a_{\mathrm{ij}}b_{\mathrm{ij}}}-\left(2-P_{\mathrm{ab}}\right)\left[S_{a_{\mathrm{mm}}}^{a_{\mathrm{ij}}}\lambda_{m}^{a_{\mathrm{mm}}}{\mathrm{K̅}}_{\mathrm{ij}}^{mb_{\mathrm{ij}}}\right]+\left(2-P_{\mathrm{ab}}\right)\lambda_{i}^{a_{\mathrm{ii}}}S_{a_{\mathrm{ii}}}^{a_{\mathrm{ij}}}{\mathrm{F̃}}_{jb_{\mathrm{ij}}}$$

The λ_2_ contributions to *l*_*ij*_^*a*_*ij*_ *b*_*ij*_^, the terms that arise from λ_*mn*_^*e*_*mn*_ *f*_*mn*_^∂*R*_*mn*_^*e*_*mn*_ *f*_*mn*_^/∂*T*_*ij*_^*a*_*ij*_ *b*_*ij*_^, contain the following intermediates. First, we present the intermediates that have been derived previously^82^

76 $$\beta_{\mathrm{ij}}^{\mathrm{kl}}={\mathrm{B̃}}_{\mathrm{ki}}^{Q_{\mathrm{ij}}}{\mathrm{B̃}}_{\mathrm{lj}}^{Q_{\mathrm{ij}}}+T_{\mathrm{ij}}^{c_{\mathrm{ij}}d_{\mathrm{ij}}}B_{kc_{\mathrm{ij}}}^{Q_{\mathrm{ij}}}B_{ld_{\mathrm{ij}}}^{Q_{\mathrm{ij}}}$$

77 $$\gamma_{\mathrm{ki}}^{a_{\mathrm{ki}}c_{\mathrm{ki}}}=-{\mathrm{T̃}}_{l}^{a_{\mathrm{ki}}}J_{\mathrm{ki}}^{lc_{\mathrm{ki}}}+{\mathrm{T̃}}_{i}^{b_{\mathrm{ki}}}J_{kb_{\mathrm{ki}}}^{a_{\mathrm{ki}}c_{\mathrm{ki}}}-{\mathrm{T̃}}_{i}^{b_{\mathrm{kl}}}K_{\mathrm{kl}}^{b_{\mathrm{kl}}c_{\mathrm{kl}}}S_{c_{\mathrm{kl}}}^{c_{\mathrm{ki}}}{\mathrm{T̃}}_{l}^{a_{\mathrm{ki}}}-\frac{1}{2}S_{a_{\mathrm{li}}}^{a_{\mathrm{ki}}}T_{\mathrm{li}}^{a_{\mathrm{li}}d_{\mathrm{li}}}S_{d_{\mathrm{kl}}}^{d_{\mathrm{li}}}K_{\mathrm{kl}}^{d_{\mathrm{kl}}c_{\mathrm{kl}}}S_{c_{\mathrm{kl}}}^{c_{\mathrm{ki}}}$$

78 $$\delta_{\mathrm{ik}}^{a_{\mathrm{ik}}c_{\mathrm{ik}}}=-{\mathrm{T̃}}_{l}^{a_{\mathrm{ik}}}M_{\mathrm{ik}}^{lc_{\mathrm{ik}}}+{\mathrm{T̃}}_{i}^{b_{\mathrm{ik}}}M_{kb_{\mathrm{ki}}}^{c_{\mathrm{ki}}a_{\mathrm{ki}}}-{\mathrm{T̃}}_{i}^{b_{\mathrm{lk}}}L_{\mathrm{lk}}^{b_{\mathrm{lk}}c_{\mathrm{lk}}}S_{c_{\mathrm{lk}}}^{c_{\mathrm{ki}}}{\mathrm{T̃}}_{l}^{a_{\mathrm{ik}}}+\frac{1}{2}S_{a_{\mathrm{il}}}^{a_{\mathrm{ik}}}U_{\mathrm{il}}^{a_{\mathrm{il}}d_{\mathrm{il}}}S_{d_{\mathrm{kl}}}^{d_{\mathrm{il}}}L_{\mathrm{kl}}^{c_{\mathrm{kl}}d_{\mathrm{kl}}}S_{c_{\mathrm{kl}}}^{c_{\mathrm{ik}}}$$

79 $${\tilde{\mathrm{F̃}}}_{b_{\mathrm{ij}}c_{\mathrm{ij}}}={\mathrm{F̃}}_{b_{\mathrm{ij}}c_{\mathrm{ij}}}-S_{b_{\mathrm{kl}}}^{b_{\mathrm{ij}}}U_{\mathrm{kl}}^{b_{\mathrm{kl}}d_{\mathrm{kl}}}K_{\mathrm{kl}}^{c_{\mathrm{kl}}d_{\mathrm{kl}}}S_{c_{\mathrm{kl}}}^{c_{\mathrm{ij}}}$$

80 $${\tilde{\mathrm{F̃}}}_{\mathrm{kj}}={\mathrm{F̃}}_{\mathrm{kj}}+\left(S_{c_{\mathrm{lj}}}^{c_{\mathrm{lk}}}U_{\mathrm{lj}}^{c_{\mathrm{lj}}d_{\mathrm{lj}}}S_{d_{\mathrm{lj}}}^{d_{\mathrm{lk}}}\right)K_{\mathrm{lk}}^{c_{\mathrm{lk}}d_{\mathrm{lk}}}$$

Next, we present some new intermediates derived from these intermediates that are used in this work

81 $$\alpha_{\mathrm{ij}}^{\mathrm{kl}}=\lambda_{\mathrm{ij}}^{e_{\mathrm{ij}}f_{\mathrm{ij}}}\left(S_{e_{\mathrm{kl}}}^{e_{\mathrm{ij}}}T_{\mathrm{kl}}^{e_{\mathrm{kl}}f_{\mathrm{kl}}}S_{f_{\mathrm{kl}}}^{f_{\mathrm{ij}}}\right)$$

82 $${\mathrm{\gamma ̃}}_{\mathrm{in}}^{f_{\mathrm{jn}}b_{\mathrm{ij}}}=S_{f_{\mathrm{in}}}^{f_{\mathrm{jn}}}\gamma_{\mathrm{in}}^{f_{\mathrm{in}}b_{\mathrm{in}}}S_{b_{\mathrm{in}}}^{b_{\mathrm{ij}}}+J_{\mathrm{in}}^{f_{\mathrm{jn}}b_{\mathrm{ij}}}$$

83 $${\mathrm{\delta ̃}}_{\mathrm{nj}}^{f_{\mathrm{ni}}b_{\mathrm{ij}}}=S_{f_{\mathrm{nj}}}^{f_{\mathrm{ni}}}\delta_{\mathrm{nj}}^{f_{\mathrm{nj}}b_{\mathrm{nj}}}S_{b_{\mathrm{nj}}}^{b_{\mathrm{ij}}}+\left(2K_{\mathrm{nj}}^{f_{\mathrm{ni}}b_{\mathrm{ij}}}-J_{\mathrm{nj}}^{f_{\mathrm{ni}}b_{\mathrm{ij}}}\right)$$

84 $${\tilde{\mathrm{\gamma ̃}}}_{\mathrm{in}}^{f_{\mathrm{nj}}b_{\mathrm{ij}}}=\left(S_{f_{\mathrm{kn}}}^{f_{\mathrm{jn}}}T_{\mathrm{kn}}^{f_{\mathrm{kn}}c_{\mathrm{kn}}}S_{c_{\mathrm{kn}}}^{c_{\mathrm{kj}}}\right)\left(S_{c_{\mathrm{ik}}}^{c_{\mathrm{kj}}}K_{\mathrm{ik}}^{c_{\mathrm{ik}}b_{\mathrm{ik}}}S_{b_{\mathrm{ik}}}^{b_{\mathrm{ij}}}\right)$$

85 $${\tilde{\mathrm{\delta ̃}}}_{\mathrm{nj}}^{f_{\mathrm{in}}b_{\mathrm{ij}}}=\left(S_{f_{\mathrm{nk}}}^{f_{\mathrm{ni}}}U_{\mathrm{nk}}^{f_{\mathrm{nk}}c_{\mathrm{nk}}}S_{c_{\mathrm{ik}}}^{c_{\mathrm{nk}}}\right)\left(S_{c_{\mathrm{kj}}}^{c_{\mathrm{ik}}}L_{\mathrm{kj}}^{c_{\mathrm{kj}}b_{\mathrm{kj}}}S_{b_{\mathrm{ij}}}^{b_{\mathrm{kj}}}\right)$$

Finally, the λ_2_ contributions to *l*_*ij*_^*a*_*ij*_ *b*_*ij*_^ take the form

86 $${\mathrm{l̅}}_{\mathrm{ij}}^{a_{\mathrm{ij}}b_{\mathrm{ij}}}+=\frac{1}{2}\left[\lambda_{\mathrm{ij}}^{e_{\mathrm{ij}}f_{\mathrm{ij}}}{\mathrm{B̃}}_{e_{\mathrm{ij}}a_{\mathrm{ij}}}^{Q_{\mathrm{ij}}}{\mathrm{B̃}}_{f_{\mathrm{ij}}b_{\mathrm{ij}}}^{Q_{\mathrm{ij}}}+\alpha_{\mathrm{ij}}^{\mathrm{kl}}B_{ka_{\mathrm{ij}}}^{Q_{\mathrm{ij}}}B_{lb_{\mathrm{ij}}}^{Q_{\mathrm{ij}}}\right]$$

87 $${\mathrm{l̅}}_{\mathrm{ij}}^{a_{\mathrm{ij}}b_{\mathrm{ij}}}+=\frac{1}{2}\left(S_{a_{\mathrm{mn}}}^{a_{\mathrm{ij}}}\lambda_{\mathrm{mn}}^{a_{\mathrm{mn}}b_{\mathrm{mn}}}S_{b_{\mathrm{mn}}}^{b_{\mathrm{ij}}}\right)\beta_{\mathrm{mn}}^{\mathrm{ij}}$$

88 $${\mathrm{l̅}}_{\mathrm{ij}}^{a_{\mathrm{ij}}b_{\mathrm{ij}}}+=-S_{a_{\mathrm{jn}}}^{a_{\mathrm{ij}}}{\mathrm{\lambda ̅}}_{\mathrm{jn}}^{a_{\mathrm{jn}}f_{\mathrm{jn}}}{\mathrm{\gamma ̃}}_{\mathrm{in}}^{f_{\mathrm{jn}}b_{\mathrm{ij}}}+\frac{1}{2}S_{a_{\mathrm{kj}}}^{a_{\mathrm{ij}}}\left(S_{a_{\mathrm{jn}}}^{a_{\mathrm{kj}}}{\mathrm{\lambda ̅}}_{\mathrm{jn}}^{a_{\mathrm{jn}}f_{\mathrm{jn}}}{\tilde{\mathrm{\gamma ̃}}}_{\mathrm{in}}^{f_{\mathrm{nj}}b_{\mathrm{ij}}}\right)$$

89 $${\mathrm{l̅}}_{\mathrm{ij}}^{a_{\mathrm{ij}}b_{\mathrm{ij}}}+=\left(2-P_{\mathrm{ab}}\right)\left[\frac{1}{2}S_{a_{\mathrm{ij}}}^{a_{\mathrm{in}}}\lambda_{\mathrm{in}}^{a_{\mathrm{in}}f_{\mathrm{in}}}{\mathrm{\delta ̃}}_{\mathrm{nj}}^{f_{\mathrm{ni}}b_{\mathrm{ij}}}+\frac{1}{4}S_{a_{\mathrm{ik}}}^{a_{\mathrm{ij}}}\left(S_{a_{\mathrm{in}}}^{a_{\mathrm{ik}}}\lambda_{\mathrm{in}}^{a_{\mathrm{in}}f_{\mathrm{in}}}{\tilde{\mathrm{\delta ̃}}}_{\mathrm{nj}}^{f_{\mathrm{in}}b_{\mathrm{ij}}}\right)\right]$$

90 $${\mathrm{l̅}}_{\mathrm{ij}}^{a_{\mathrm{ij}}b_{\mathrm{ij}}}+=\lambda_{\mathrm{ij}}^{a_{\mathrm{ij}}f_{\mathrm{ij}}}{\tilde{\mathrm{F̃}}}_{f_{\mathrm{ij}}b_{\mathrm{ij}}}-\left(2-P_{\mathrm{ab}}\right)\rho_{a_{\mathrm{mn}}c_{\mathrm{mn}}}^{\mathrm{VV}}S_{a_{\mathrm{ij}}}^{a_{\mathrm{mn}}}K_{\mathrm{ij}}^{c_{\mathrm{ij}}b_{\mathrm{ij}}}S_{c_{\mathrm{ij}}}^{c_{\mathrm{mn}}}$$

91 $${\mathrm{l̅}}_{\mathrm{ij}}^{a_{\mathrm{ij}}b_{\mathrm{ij}}}-=\left(S_{a_{\mathrm{in}}}^{a_{\mathrm{ij}}}\lambda_{\mathrm{in}}^{a_{\mathrm{in}}b_{\mathrm{in}}}S_{b_{\mathrm{in}}}^{b_{\mathrm{ij}}}\right){\tilde{\mathrm{F̃}}}_{\mathrm{jn}}+\rho_{\mathrm{jk}}^{\mathrm{OO}}\left(S_{a_{\mathrm{ij}}}^{a_{\mathrm{ik}}}L_{\mathrm{ik}}^{a_{\mathrm{ik}}b_{\mathrm{ik}}}S_{b_{\mathrm{ij}}}^{b_{\mathrm{ik}}}\right)$$

where *l̅* _*ij*_^*a*_*ij*_ *b*_*ij*_^ is the nonsymmetrized λ_2_ residual, and the full residual is defined as

92 $$l_{\mathrm{ij}}^{a_{\mathrm{ij}}b_{\mathrm{ij}}}={\mathrm{l̅}}_{\mathrm{ij}}^{a_{\mathrm{ij}}b_{\mathrm{ij}}}+{\mathrm{l̅}}_{\mathrm{ji}}^{b_{\mathrm{ij}}a_{\mathrm{ij}}}$$

### IV.E Lambda (or Asymmetric) Triples Correction

We now present the equation for the asymmetric triples correction or (*T*)_Λ_ in the local pair natural orbital basis. First, we define the *W* intermediate from DLPNO–CCSD­(*T*)^82^

93 $$W_{\mathrm{ijk}}^{a_{\mathrm{ijk}}b_{\mathrm{ijk}}c_{\mathrm{ijk}}}=P_{L}\left[\left(ia_{\mathrm{ijk}}|b_{\mathrm{ijk}}d_{\mathrm{ijk}}\right)S_{c_{\mathrm{kj}}}^{c_{\mathrm{ijk}}}T_{\mathrm{kj}}^{c_{\mathrm{kj}}d_{\mathrm{kj}}}S_{d_{\mathrm{kj}}}^{d_{\mathrm{ijk}}}-\left(\mathrm{jl}|kc_{\mathrm{ijk}}\right)S_{a_{\mathrm{il}}}^{a_{\mathrm{ijk}}}T_{\mathrm{il}}^{a_{\mathrm{il}}b_{\mathrm{il}}}S_{b_{\mathrm{il}}}^{b_{\mathrm{ijk}}}\right]$$

Once *W*_*ijk*_^*a*_*ijk*_ *b*_*ijk*_ *c*_*ijk*_^ is initially formed, the (*T*)_Λ_ energy is iteratively solved for by accounting for the contribution of the off-diagonal LMO Fock matrix elements, which only arise from the of noncanonical HF orbitals (LMOs). With

94 $$R_{\mathrm{ijk}}^{a_{\mathrm{ijk}}b_{\mathrm{ijk}}c_{\mathrm{ijk}}}=W_{\mathrm{ijk}}^{a_{\mathrm{ijk}}b_{\mathrm{ijk}}c_{\mathrm{ijk}}}-T_{\mathrm{ijk}}^{a_{\mathrm{ijk}}b_{\mathrm{ijk}}c_{\mathrm{ijk}}}\left(\epsilon_{a_{\mathrm{ijk}}}+\epsilon_{b_{\mathrm{ijk}}}+\epsilon_{c_{\mathrm{ijk}}}-f_{\mathrm{ii}}-f_{\mathrm{jj}}-f_{\mathrm{kk}}\right)-\sum_{l\neq i}f_{\mathrm{il}}T_{\mathrm{ljk}}^{a_{\mathrm{ljk}}b_{\mathrm{ljk}}c_{\mathrm{ljk}}}S_{a_{\mathrm{ljk}}b_{\mathrm{ljk}}c_{\mathrm{ljk}}}^{a_{\mathrm{ijk}}b_{\mathrm{ijk}}c_{\mathrm{ijk}}}-\sum_{l\neq j}f_{\mathrm{jl}}T_{\mathrm{ilk}}^{a_{\mathrm{ilk}}b_{\mathrm{ilk}}c_{\mathrm{ilk}}}S_{a_{\mathrm{ilk}}b_{\mathrm{ilk}}c_{\mathrm{ilk}}}^{a_{\mathrm{ijk}}b_{\mathrm{ijk}}c_{\mathrm{ijk}}}-\sum_{l\neq k}f_{\mathrm{kl}}T_{\mathrm{ijl}}^{a_{\mathrm{ijl}}b_{\mathrm{ijl}}c_{\mathrm{ijl}}}S_{a_{\mathrm{ijl}}b_{\mathrm{ajl}}c_{\mathrm{ajl}}}^{a_{\mathrm{ijk}}b_{\mathrm{ijk}}c_{\mathrm{ijk}}}$$

the triples amplitudes are iteratively updated through

95 $$T_{\mathrm{ijk}}^{a_{\mathrm{ijk}}b_{\mathrm{ijk}}c_{\mathrm{ijk}}}-=\frac{R_{\mathrm{ijk}}^{a_{\mathrm{ijk}}b_{\mathrm{ijk}}c_{\mathrm{ijk}}}}{\epsilon_{a_{\mathrm{ijk}}}+\epsilon_{b_{\mathrm{ijk}}}+\epsilon_{c_{\mathrm{ijk}}}-F_{\mathrm{ii}}-F_{\mathrm{jj}}-F_{\mathrm{kk}}}$$

In each iteration, the DLPNO–CCSD­(T)_Λ_ energy is computed through the following “connected” (c) and “disconnected” (d) contributions.

96 $$\Lambda_{\mathrm{ijk}}^{a_{\mathrm{ijk}}b_{\mathrm{ijk}}c_{\mathrm{ijk}}}\left(c\right)=P_{L}\left[\left(ia_{\mathrm{ijk}}|b_{\mathrm{ijk}}d_{\mathrm{ijk}}\right)S_{c_{\mathrm{kj}}}^{c_{\mathrm{ijk}}}\lambda_{\mathrm{kj}}^{c_{\mathrm{kj}}d_{\mathrm{kj}}\prime }S_{d_{\mathrm{kj}}}^{d_{\mathrm{ijk}}}-\left(\mathrm{jl}|kc_{\mathrm{ijk}}\right)S_{a_{\mathrm{il}}}^{a_{\mathrm{ijk}}}\lambda_{\mathrm{il}}^{a_{\mathrm{il}}b_{\mathrm{il}}\prime }S_{b_{\mathrm{il}}}^{b_{\mathrm{ijk}}}\right]$$

97 $$\Lambda_{\mathrm{ijk}}^{a_{\mathrm{ijk}}b_{\mathrm{ijk}}c_{\mathrm{ijk}}}\left(d\right)=P_{S}\left[\lambda_{i}^{a_{\mathrm{ii}}\prime }S_{a_{\mathrm{ii}}}^{a_{\mathrm{ijk}}}\left(jb_{\mathrm{ijk}}|kc_{\mathrm{ijk}}\right)\right]$$

One thing to note is that the λ amplitudes, as defined here, are different with respect to the ones directly solved for in our equations. The amplitudes solved in our equations are presented in the “contra-variant spin-summed” basis, whereas the ones expected here are not in that basis, and they need to be “re-normalized”

98 $$\lambda_{i}^{a_{\mathrm{ii}}\prime }=\frac{1}{2}\lambda_{i}^{a_{\mathrm{ii}}}$$

99 $$\lambda_{\mathrm{ij}}^{a_{\mathrm{ij}}b_{\mathrm{ij}}\prime }=\frac{1}{3}\lambda_{\mathrm{ij}}^{a_{\mathrm{ij}}b_{\mathrm{ij}}}+\frac{1}{6}\lambda_{\mathrm{ij}}^{b_{\mathrm{ij}}a_{\mathrm{ij}}}$$

Thus, the DLPNO–CCSD­(T)_Λ_ energy expression takes the form

100 $$\Lambda_{\mathrm{ijk}}^{a_{\mathrm{ijk}}b_{\mathrm{ijk}}c_{\mathrm{ijk}}}=\Lambda_{\mathrm{ijk}}^{a_{\mathrm{ijk}}b_{\mathrm{ijk}}c_{\mathrm{ijk}}}\left(c\right)+\Lambda_{\mathrm{ijk}}^{a_{\mathrm{ijk}}b_{\mathrm{ijk}}c_{\mathrm{ijk}}}\left(d\right)$$

101 $$E_{{\left(T\right)}_{\Lambda }}=\sum_{i\leq j\leq k}\frac{T_{\mathrm{ijk}}^{a_{\mathrm{ijk}}b_{\mathrm{ijk}}c_{\mathrm{ijk}}}}{1+\left(\delta_{\mathrm{ij}}+\delta_{\mathrm{jk}}+\delta_{\mathrm{ik}}\right)+2\delta_{\mathrm{ij}}\delta_{\mathrm{jk}}\delta_{\mathrm{ik}}}\times \left(8\Lambda_{\mathrm{ijk}}^{a_{\mathrm{ijk}}b_{\mathrm{ijk}}c_{\mathrm{ijk}}}-4\Lambda_{\mathrm{ijk}}^{b_{\mathrm{ijk}}a_{\mathrm{ijk}}c_{\mathrm{ijk}}}-4\Lambda_{\mathrm{ijk}}^{a_{\mathrm{ijk}}c_{\mathrm{ijk}}b_{\mathrm{ijk}}}-4\Lambda_{\mathrm{ijk}}^{c_{\mathrm{ijk}}a_{\mathrm{ijk}}b_{\mathrm{ijk}}}+2\Lambda_{\mathrm{ijk}}^{b_{\mathrm{ijk}}c_{\mathrm{ijk}}a_{\mathrm{ijk}}}+2\Lambda_{\mathrm{ijk}}^{c_{\mathrm{ijk}}a_{\mathrm{ijk}}b_{\mathrm{ijk}}}\right)$$

Note that this takes a different form than eq 23 since we are taking a sum over restricted *i* ≤ *j* ≤ *k* to conserve memory.

## V Implementation Details

### V.A CCSD Λ Algorithm Details

Some of our equations are implemented slightly differently than how they are derived, due to accuracy and efficiency considerations. Many “double projections” such as the one in eq 84 are removed for the sake of accuracy and numerical stability. Thus, eq 84 changes from

102 $${\tilde{\mathrm{\gamma ̃}}}_{\mathrm{in}}^{f_{\mathrm{nj}}b_{\mathrm{ij}}}=\left(S_{f_{\mathrm{kn}}}^{f_{\mathrm{jn}}}T_{\mathrm{kn}}^{f_{\mathrm{kn}}c_{\mathrm{kn}}}S_{c_{\mathrm{kn}}}^{c_{\mathrm{kj}}}\right)\left(S_{c_{\mathrm{ik}}}^{c_{\mathrm{kj}}}K_{\mathrm{ik}}^{c_{\mathrm{ik}}b_{\mathrm{ik}}}S_{b_{\mathrm{ik}}}^{b_{\mathrm{ij}}}\right)$$

to

103 $${\tilde{\mathrm{\gamma ̃}}}_{\mathrm{in}}^{f_{\mathrm{nj}}b_{\mathrm{ij}}}=\left(S_{f_{\mathrm{kn}}}^{f_{\mathrm{jn}}}T_{\mathrm{kn}}^{f_{\mathrm{kn}}c_{\mathrm{kn}}}S_{c_{\mathrm{kn}}}^{c_{\mathrm{ik}}}\right)\left(K_{\mathrm{ik}}^{c_{\mathrm{ik}}b_{\mathrm{ik}}}S_{b_{\mathrm{ik}}}^{b_{\mathrm{ij}}}\right)$$

where we remove the double projection *S*_*c*_*kn*__^*c*_*kj*_^ *S*_*c*_*ik*__^*c*_*kj*_^ and replaced it with *S*_*c*_*ik*__^*c*_*kn*_^. Similarly, eq 104 changes from

104 $${\tilde{\mathrm{\delta ̃}}}_{\mathrm{nj}}^{f_{\mathrm{in}}b_{\mathrm{ij}}}=\left(S_{f_{\mathrm{nk}}}^{f_{\mathrm{ni}}}U_{\mathrm{nk}}^{f_{\mathrm{nk}}c_{\mathrm{nk}}}S_{c_{\mathrm{ik}}}^{c_{\mathrm{nk}}}\right)\left(S_{c_{\mathrm{kj}}}^{c_{\mathrm{ik}}}L_{\mathrm{kj}}^{c_{\mathrm{kj}}b_{\mathrm{kj}}}S_{b_{\mathrm{ij}}}^{b_{\mathrm{kj}}}\right)$$

to

$${\overset{\sim }{\mathrm{\delta ̃}}}_{\mathrm{nj}}^{f_{\mathrm{in}}b_{\mathrm{ij}}}=\left(S_{f_{\mathrm{nk}}}^{f_{\mathrm{ni}}}U_{\mathrm{nk}}^{f_{\mathrm{nk}}c_{\mathrm{nk}}}S_{c_{\mathrm{kj}}}^{c_{\mathrm{nk}}}\right)\left(L_{\mathrm{kj}}^{c_{\mathrm{kj}}b_{\mathrm{kj}}}S_{b_{\mathrm{ij}}}^{b_{\mathrm{kj}}}\right)$$ 105

where we replaced *S*_*c*_*ik*__^*c*_*nk*_^ *S*_*c*_*kj*__^*c*_*ik*_^ with *S*_*c*_*kj*__^*c*_*nk*_^. In addition, eqs 88 and 89 become, by respectively changing *S*_*a*_*kj*__^*a*_*ij*_^ *S*_*a*_*jn*__^*a*_*kj*_^ → *S*_*a*_*jn*__^*a*_*ij*_^ and *S*_*a*_*ik*__^*a*_*ij*_^ *S*_*a*_*in*__^*a*_*ik*_^ → *S*_*a*_*in*__^*a*_*ij*_^.

106 $${\mathrm{l̅}}_{\mathrm{ij}}^{a_{\mathrm{ij}}b_{\mathrm{ij}}}+=-S_{a_{\mathrm{jn}}}^{a_{\mathrm{ij}}}{\mathrm{\lambda ̅}}_{\mathrm{jn}}^{a_{\mathrm{jn}}f_{\mathrm{jn}}}{\mathrm{\gamma ̃}}_{\mathrm{in}}^{f_{\mathrm{jn}}b_{\mathrm{ij}}}+\frac{1}{2}S_{a_{\mathrm{jn}}}^{a_{\mathrm{ij}}}{\mathrm{\lambda ̅}}_{\mathrm{jn}}^{a_{\mathrm{jn}}f_{\mathrm{jn}}}{\tilde{\mathrm{\gamma ̃}}}_{\mathrm{in}}^{f_{\mathrm{nj}}b_{\mathrm{ij}}}$$

107 $${\mathrm{l̅}}_{\mathrm{ij}}^{a_{\mathrm{ij}}b_{\mathrm{ij}}}+=\left(2-P_{\mathrm{ab}}\right)\left[\frac{1}{2}S_{a_{\mathrm{ij}}}^{a_{\mathrm{in}}}\lambda_{\mathrm{in}}^{a_{\mathrm{in}}f_{\mathrm{in}}}{\mathrm{\delta ̃}}_{\mathrm{nj}}^{f_{\mathrm{ni}}b_{\mathrm{ij}}}+\frac{1}{4}S_{a_{\mathrm{in}}}^{a_{\mathrm{ij}}}\lambda_{\mathrm{in}}^{a_{\mathrm{in}}f_{\mathrm{in}}}{\tilde{\mathrm{\delta ̃}}}_{\mathrm{nj}}^{f_{\mathrm{in}}b_{\mathrm{ij}}}\right]$$

With eqs 86, 87, and 90, since they involve expensive operations, they are only computed with restricted indexing *i* ≤ *j*, thus, they are symmetrized and their contributions added to *l*_*ij*_^*a*_*ij*_ *b*_*ij*_^ directly and not *l̅*_*ij*_^*a*_*ij*_ *b*_ij_^

108 $$A_{\mathrm{ij}}^{a_{\mathrm{ij}}b_{\mathrm{ij}}}=\lambda_{\mathrm{ij}}^{e_{\mathrm{ij}}f_{\mathrm{ij}}}{\mathrm{B̃}}_{e_{\mathrm{ij}}a_{\mathrm{ij}}}^{Q_{\mathrm{ij}}}{\mathrm{B̃}}_{f_{\mathrm{ij}}b_{\mathrm{ij}}}^{Q_{\mathrm{ij}}}+\alpha_{\mathrm{ij}}^{\mathrm{kl}}B_{ka_{\mathrm{ij}}}^{Q_{\mathrm{ij}}}B_{lb_{\mathrm{ij}}}^{Q_{\mathrm{ij}}}$$

109 $$B_{\mathrm{ij}}^{a_{\mathrm{ij}}b_{\mathrm{ij}}}=\left(S_{a_{\mathrm{mn}}}^{a_{\mathrm{ij}}}\lambda_{\mathrm{mn}}^{a_{\mathrm{mn}}b_{\mathrm{mn}}}S_{b_{\mathrm{mn}}}^{b_{\mathrm{ij}}}\right)\beta_{\mathrm{mn}}^{\mathrm{ij}}$$

110 $$E_{\mathrm{ij}}^{a_{\mathrm{ij}}b_{\mathrm{ij}}}=\lambda_{\mathrm{ij}}^{a_{\mathrm{ij}}f_{\mathrm{ij}}}{\tilde{\mathrm{F̃}}}_{f_{\mathrm{ij}}b_{\mathrm{ij}}}+{\tilde{\mathrm{F̃}}}_{f_{\mathrm{ij}}a_{\mathrm{ij}}}\lambda_{\mathrm{ij}}^{f_{\mathrm{ij}}b_{\mathrm{ij}}}-\left[S_{a_{\mathrm{ij}}}^{a_{\mathrm{mn}}}\rho_{a_{\mathrm{mn}}c_{\mathrm{mn}}}^{\mathrm{VV}}S_{c_{\mathrm{ij}}}^{c_{\mathrm{mn}}}\right]L_{\mathrm{ij}}^{c_{\mathrm{ij}}b_{\mathrm{ij}}}-L_{\mathrm{ij}}^{a_{\mathrm{ij}}c_{\mathrm{ij}}}\left[S_{b_{\mathrm{ij}}}^{b_{\mathrm{mn}}}\rho_{b_{\mathrm{mn}}c_{\mathrm{mn}}}^{\mathrm{VV}}S_{c_{\mathrm{ij}}}^{c_{\mathrm{mn}}}\right]$$

After these intermediates are computed,

111 $$l_{\mathrm{ij}}^{a_{\mathrm{ij}}b_{\mathrm{ij}}}+=A_{\mathrm{ij}}^{a_{\mathrm{ij}}b_{\mathrm{ij}}}+B_{\mathrm{ij}}^{a_{\mathrm{ij}}b_{\mathrm{ij}}}+E_{\mathrm{ij}}^{a_{\mathrm{ij}}b_{\mathrm{ij}}}$$

112 $$l_{\mathrm{ji}}^{b_{\mathrm{ij}}a_{\mathrm{ij}}}+=A_{\mathrm{ij}}^{b_{\mathrm{ij}}a_{\mathrm{ij}}}+B_{\mathrm{ij}}^{b_{\mathrm{ij}}a_{\mathrm{ij}}}+E_{\mathrm{ij}}^{b_{\mathrm{ij}}a_{\mathrm{ij}}}$$

In addition, we do not directly form the *F̂*_*f*_*mn*_ *c*_*mn*__^*ia*_*mn*_^ intermediate (eq 65), due to memory and runtime considerations. Therefore, as implemented in our code, the first term of eq 68 now becomes

113 $$l_{i}^{a_{\mathrm{ii}}}+=\sum_{\mathrm{mn}}\left[\sum_{Q_{\mathrm{mn}}}2\left({\mathrm{B̃}}_{f_{\mathrm{mn}}c_{\mathrm{mn}}}^{Q_{\mathrm{mn}}}\rho_{f_{\mathrm{mn}}c_{\mathrm{mn}}}^{\mathrm{VV}}\right)B_{ia_{\mathrm{mn}}}^{Q_{\mathrm{mn}}}-\left({\mathrm{B̃}}_{f_{\mathrm{mn}}a_{\mathrm{mn}}}^{Q_{\mathrm{mn}}}\rho_{f_{\mathrm{mn}}c_{\mathrm{mn}}}^{\mathrm{VV}}B_{ic_{\mathrm{mn}}}^{Q_{\mathrm{mn}}}\right)\right]S_{a_{\mathrm{ii}}}^{a_{\mathrm{mn}}}$$

### V.B (*T*)_Λ_ Algorithm Details

In our DLPNO–CCSD­(T)_Λ_ algorithm, all pairs that would have been considered “MP2 weak pairs” in our prior work^82^ will be treated at the CCSD level of theory, as opposed to traditional implementations of DLPNO–CCSD­(T). Apart from this, our DLPNO–CCSD­(T)_Λ_ method follows an analogous procedure as DLPNO–CCSD­(T), with the initial prescreening of triplets now performed using semicanonical DLPNO–CCSD­(T0)_Λ_ instead of DLPNO–CCSD­(T0), where DLPNO–CCSD­(T0)_Λ_ denotes the energy computed with eq 101 without updating the triples amplitude with contributions from off-diagonal Fock matrix elements. Thus, the final DLPNO–CCSD­(T)_Λ_ energy takes a similar form to the final DLPNO–CCSD­(T) energy

114 $$E_{\mathrm{DLPNO}‐{\left(T\right)}_{\Lambda }}=\sum_{i\leq j\leq k}E_{{\left(T0\right)}_{\Lambda }}^{\mathrm{ijk}}\left[T_{\mathrm{CUT}\_\mathrm{TNO}}\right]+\sum_{\mathrm{ijk}\in \mathrm{strong}\;\mathrm{triplets}}\left(E_{{\left(T\right)}_{\Lambda }}^{\mathrm{ijk}}-E_{{\left(T0\right)}_{\Lambda }}^{\mathrm{ijk}}\right)\left[T_{\mathrm{CUT}\_\mathrm{TNO}}\times T_{\mathrm{STRONG}\_\mathrm{SCALE}}\right]+\sum_{\mathrm{ijk}\in \mathrm{weak}\;\mathrm{triplets}}\left(E_{{\left(T\right)}_{\Lambda }}^{\mathrm{ijk}}-E_{{\left(T0\right)}_{\Lambda }}^{\mathrm{ijk}}\right)\left[T_{\mathrm{CUT}\_\mathrm{TNO}}\times T_{\mathrm{WEAK}\_\mathrm{SCALE}}\right]+\sum_{\mathrm{ijk}\in \mathrm{screened}\;\mathrm{triplets}}\Delta E_{{\left(T0\right)}_{\Lambda }}^{\mathrm{ijk}}\left[T_{\mathrm{CUT}\_\mathrm{TNO}\_\mathrm{PRE}}\right]$$

Similar to DLPNO–CCSD­(T), the contribution from our screened triplets are computed noniteratively with TNO tolerance *T*_CUT_TNO_PRE_, typically 10^–7^. A noniterative DLPNO–CCSD­(T0)_Λ_ energy is computed with a tight tolerance *T*_CUT_TNO_, typically 10^–9^, where strong triplets are defined as the triplets that account for 90% of the triples energy, and weak triplets form the rest of them. Finally, the off-diagonal couplings are computed with iterative (*T*)_Λ_, with *T*_STRONG_SCALE_ = 10 and *T*_WEAK_SCALE_ = 100, by default. For more details on this procedure, the reader is referred to our prior work.^82^ In addition, when DLPNO–CCSD­(T) energies are referenced in the results, it refers to the DLPNO–CCSD­(T) energies computed under the same framework as DLPNO–CCSD­(T)_Λ_. This means all weak pairs become strong pairs, and the prescreening for triplets as well as determination of strong and weak triplets involve DLPNO–CCSD­(T0)_Λ_ and not DLPNO–CCSD­(T0) energetics. Unless otherwise stated, all canonical CCSD­(T)_Λ_ utilize the density-fitted variant^50^ implemented in the Psi4 package.^102^ Formally, DLPNO–CCSD­(T)_Λ_ has the same cost as DLPNO–CCSD­(T), being affordable for the same systems in which DLPNO–CCSD­(T) is possible, pending further code optimization. The storage requirements are similar, only requiring the storage of the additional Λ_*ijk*_^*a*_*ijk*_ *b*_*ijk*_ *c*_*ijk*_^ intermediate, in addition to *W*_*ijk*_^*a*_*ijk*_ *b*_*ijk*_ *c*_*ijk*_^, *V*_*ijk*_^*a*_*ijk*_ *b*_*ijk*_ *c*_*ijk*_^, and *T*_*ijk*_^*a*_*ijk*_ *b*_*ijk*_ *c*_*ijk*_^ from DLPNO–CCSD­(T). In terms of runtime, the rate-limiting step is the iterative update of *T*_*ijk*_^*a*_*ijk*_ *b*_*ijk*_ *c*_*ijk*_^, so the runtime of DLPNO–CCSD­(T)_Λ_ would be very similar to DLPNO–CCSD­(T), with additional time needed to solve for λ_*i*_^*a*_*ii*_^ and λ_*ij*_^*a*_*ij*_ *b*_*ij*_^.

### V.C Computational Details

For all correlated computations, the frozen-core approximation is used, that is, all nonvalence electrons are frozen. Unless otherwise stated, TightPNO convergence is used for all computations. Computations are run using AMD EPYC 9274F (Genoa) CPUs, with a base clock of 4.05 GHz and a max clock of 4.3 GHz with up to 16 CPU cores and 500 GB of RAM. Our code is available in a development branch of the freely available Psi4 program.^102^

## VI Results

### VI.A Pericyclic Reactions

A good test case for evaluating CCSD­(T)_Λ_ would be on systems with significant CCSDT-CCSD­(T) differences, such as pericyclic reactions studied in the work of Vermeeren et al.^103^ These reactions include Diels–Alder, 1,3-dipolar cycloaddition, electrocyclic rearrangement, sigmatropic rearrangement, and double group transfer prototypes. The reference geometries are given in the work of Vermeeren et al. and optimized at the CCSD­(T)/cc-pVTZ level of theory. The provided CCSDT/cc-pVDZ and CCSD­(T)/cc-pVDZ energetics are also given in that work. Results for reaction energies are presented in Table [Table tblI], while results for barrier heights are given in Table [Table tblII]. Our results indicate excellent agreement between canonical CCSD­(T)_Λ_ and DLPNO–CCSD­(T)_Λ_, as well as canonical CCSD­(T) and DLPNO–CCSD­(T), all errors being on the order of 0.2 kcal mol^–1^ or less. In addition, the corresponding DLPNO errors of CCSD­(T)_Λ_ and CCSD­(T) are effectively equivalent, the largest deviation being 0.04 kcal mol^–1^ for the Diels–Alder reaction BH2 in Table [Table tblII]. Comparing the canonical results, CCSD­(T) agrees better than CCSD­(T)_Λ_ relative to CCSDT for reaction energies in all cases, while CCSD­(T)_Λ_ agrees better than CCSD­(T) for barrier heights. This is consistent with current understanding.

**I tblI:** Pericyclic Reaction Energies for CCSD­(T)_Λ_ and CCSD­(T) Compared to CCSDT in the cc-pVDZ Basis[Table-fn tIfn1]

Reaction	Δ*E*_CCSDT_	δ_(*T*)_Λ_–*T*_	δ_DLPNO–(*T*)_Λ__	δ_(*T*)–*T*_	δ_DLPNO–(*T*)_
*trans* Diels–Alder Reaction (R1)	+24.54	–0.56	+0.05	–0.37	+0.05
Diels–Alder Reaction (R2)	+21.61	–0.52	+0.07	–0.35	+0.06
1,3-Dipolar Cycloaddition (R3)	+18.59	–0.20	+0.16	–0.11	+0.17
Electrocyclic Rearrangement (R4)	+43.64	–0.28	–0.01	–0.19	–0.01

a Canonical CCSD­(T) and CCSDT values are taken from the work of Vermeeren et al.^103^ δ_(*T*)_Λ_–*T*_ refers to the difference between canonical CCSD­(T)_Λ_ and CCSDT relative energies and δ_(*T*)–*T*_ refers to the difference between canonical CCSD­(T) and CCSDT relative energies. In addition, δ_DLPNO–(*T*)_Λ__ refers to the difference between DLPNO–CCSD­(T)_Λ_ and canonical CCSD­(T)_Λ_ energetics, and δ_DLPNO–(*T*)_ refers to the difference between DLPNO–CCSD­(T) and canonical CCSD­(T) energetics. All reported energies are in kcal mol^–1^.

**II tblII:** Pericyclic Barrier Heights for CCSD­(T)_Λ_ and CCSD­(T) Compared to CCSDT in the cc-pVDZ Basis[Table-fn tIIfn1]

Reaction	Δ*E*_CCSDT_	δ_(*T*)_Λ_–*T*_	δ_DLPNO–(*T*)_Λ__	δ_(*T*)–*T*_	δ_DLPNO–(*T*)_
*trans* Diels–Alder Reaction (BH1)	+24.54	–0.15	+0.17	–0.48	+0.14
Diels–Alder Reaction (BH2)	+21.61	–0.12	+0.19	–0.46	+0.15
1,3-Dipolar Cycloaddition (BH3)	+18.59	–0.29	+0.19	–0.70	+0.17
Electrocyclic Rearrangement (BH4)	+43.64	+0.04	+0.04	–0.16	+0.03
(*E*)-Sigmatropic Rearrangement (BH5)	+40.00	–0.10	+0.07	–0.31	+0.06
(*Z*)-Sigmatropic Rearrangement (BH6)	+37.12	–0.04	+0.09	–0.27	+0.07
Double Group Transfer (BH7)	+51.35	–0.01	+0.12	–0.30	+0.10

a Canonical CCSD­(T) and CCSDT values are taken from the work of Vermeeren et al.^103^ δ_(*T*)_Λ_–*T*_ refers to the difference between canonical CCSD­(T)_Λ_ and CCSDT relative energies and δ_(*T*)–*T*_ refers to the difference between canonical CCSD­(T) and CCSDT relative energies. In addition, δ_DLPNO–(*T*)_Λ__ refers to the difference between DLPNO–CCSD­(T)_Λ_ and canonical CCSD­(T)_Λ_ energetics, and δ_DLPNO–(*T*)_ refers to the difference between DLPNO–CCSD­(T) and canonical CCSD­(T) energetics. All reported energies are in kcal mol^–1^.

### VI.B S22 Non-Covalent Dimers

Next, we evaluate our code on a set of noncovalently bound dimers from the S22 data set.^104^ We have selected the 11 largest dimers from the set, grouped by interaction type, presented in Table [Table tblIII]. We compare canonical density-fitted CCSD­(T)_Λ_ ^50^ and canonical density-fitted CCSD­(T)^105^ results with DLPNO–CCSD­(T)_Λ_ and DLPNO–CCSD­(T), in the cc-pVDZ basis. Though the differences between (*T*) and (*T*)_Λ_ are small in most cases, largest difference being 0.11 kcal mol^–1^ for Uracil Dimer (S22–5), the DLPNO errors are consistent across both cases, the largest deviation observed being 0.02 kcal mol^–1^. This indicates that the difference between DLPNO–CCSD­(T)_Λ_ and DLPNO–CCSD­(T) effectively captures canonical (*T*)_Λ_ – (*T*) differences in spite of the magnitude of the local correlation error.

**III tblIII:** Evaluating DLPNO–CCSD­(T)_Λ_-CCSD­(T)_Λ_ and DLPNO–CCSD­(T)_Λ_-CCSD­(T)_Λ_ Errors on the 11 Largest S22 Dimers[Table-fn tIIIfn1]

Interaction	Δ*E*_CCSD(T)_Λ__	δ_DLPNO–(*T*)_Λ__	Δ*E*_CCSD(T)_	δ_DLPNO–(T)_
Uracil Dimer HB (S22–5)	–15.99	+0.14	–16.10	+0.16
2-Pyridone-2-Aminopyridine (S22–6)	–13.04	+0.07	–13.08	+0.09
Adenine-Thymine HB (S22–7)	–12.44	+0.10	–12.48	+0.12
Benzene Dimer PD (S22–11)	+0.20	+0.19	+0.17	+0.19
Pyrazine Dimer (S22–12)	–0.99	+0.16	–1.02	+0.15
Uracil Stack (S22–13)	–4.75	+0.09	–4.76	+0.09
Indole-Benzene Stack (S22–14)	–0.51	+0.24	–0.56	+0.24
Adenine-Thymine Stack (S22–15)	–5.37	+0.17	–5.39	+0.15
Benzene Dimer T-Shape (S22–20)	–1.03	+0.09	–1.04	+0.08
Indole-Benzene T-Shape (S22–21)	–3.20	+0.10	–3.21	+0.10
Phenol Dimer (S22–22)	–4.62	+0.08	–4.64	+0.08

a These are grouped into three cases: hydrogen bound (HB), dispersion dominated (DD), and mixed influence (MX), divided by a separating line. Computations performed using the cc-pVDZ basis. All reported energetics are in kcal mol^–1^.

### VI.C CH_3_I + OH^–^ Potential Energy Surface

Next, we evaluate CCSD­(T)_Λ_ on interesting points on the CH_3_I + OH^–^ *S*_*N*_2 potential energy surface, where CCSD­(T) breaks down and yields unphysical answers, from the work of Tasi, Györi, and Czakó.^45^ In Figure 1, we showcase five structures on the surface where CCSD­(T) yields significantly different energetics than CCSDT, ranked in decreasing order of CCSDT-CCSD­(T) difference. For each configuration, we report our best estimate for the CCSDTQ energetics, approximated using a composite DLPNO–CCSDTQ + (CCSDT - DLPNO–CCSDT) scheme to account for local correlation errors.^83,85^ This composite scheme is denoted as CCSDTQ*. In Figure 1, we present energetics for CCSDTQ*, CCSDT, CCSD­(T)_Λ_, and CCSD­(T). DLPNO errors for each corresponding algorithm will be given in parentheses, with the overall result for DLPNO being the canonical result combined with what is in the parentheses. These are evaluated with the aug-cc-pVDZ-PP pseudopotential basis set for iodine and aug-cc-pVDZ for other atoms. The results suggest that at such extreme geometries, CCSD­(T)_Λ_ provides qualitatively correct results, similar in performance to BCCD­(T)^45^ as reported by Tasi, Györi, and Czakó, or CCSD­(T) evaluated with Brueckner orbitals, where the coupled-cluster *T*_1_ amplitudes are set to zero through orbital optimization techniques.^106–112^

**Figure 1:**
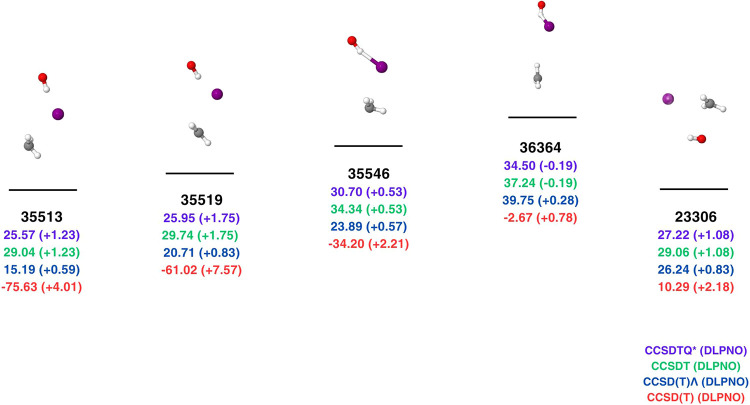
Select points along the CH_3_I + OH^–^ *S*_*N*_2 potential energy surface^45^ where CCSD­(T) provides incorrect results. The labels indicate enumerated points along surface from the original study in ref 45. Canonical CCSDT, CCSD­(T)_Λ_, and CCSD­(T) relative energies are presented, along with their DLPNO counterparts, evaluated with an aug-cc-pVDZ-PP basis for Iodine and aug-cc-pVDZ for other atoms. The best FCI estimate, CCSDTQ*, denotes DLPNO–CCSDTQ + (CCSDT – DLPNO–CCSDT). Local correlation errors with using the DLPNO versions of each method are given in parentheses. Results given in kcal mol^–1^.

The local correlation errors are mostly consistent between DLPNO–CCSDT and DLPNO–CCSD­(T)_Λ_, being on the order of 1–2 kcal mol^–1^ or less, and their magnitudes are well correlated. We find this acceptable given the slower convergence of the CC series for such geometries, as this error is less than the CCSDTQ*-CCSDT difference. However, for CCSD­(T), local correlation errors become inconsistent, though it is not unexpected given the failure of (*T*) at such configurations. These results suggest that DLPNO–CCSD­(T)_Λ_ would be a useful method to have in the development of whole potential energy surfaces, especially for larger molecules. In some cases, CCSD­(T)_Λ_ performed better than CCSDT relative to CCSDTQ*.

### VI.D (NHC)­Rh­(Cod) Catalyst Complex

We also evaluated our code on a larger rhodium-containing catalyst complex (66 atoms), recently studied by Jia et al.^113^ The catalyst, bidentate N-heterocyclic carbene (NHC)-cyclooctadiene Rh, also known as (NHC)­Rh­(Cod) is involved in the photocatalyzed visible-light-induced C–H borylation of key functional groups in organic reactions. Such a large closed-shell catalyst is a good test case for DLPNO–CCSD­(T)_Λ_, especially for the evaluation of transition states. In Figure 2, we compare DLPNO–CCSD­(T)_Λ_ with DLPNO–CCSD­(T) for the ^1^1, ^1^2, ^1^3, and ^1^4 configurations of (NHC)­Rh­(Cod), as well as their transistion states, evaluated with the cc-pVDZ basis set, and cc-pVDZ-PP for rhodium and compare them to the M06-D3/PCM free energy results presented by Jia et al.^113^ Although this is not strictly an “apples-to-apples” comparison, as solvent effects involving THF are considered in the M06-D3/PCM model, this shows that the values obtained with our algorithm are reasonable. Nonetheless, this test case is meant to be purely demonstrative, showcasing a possible application for DLPNO–CCSD­(T).

**Figure 2:**
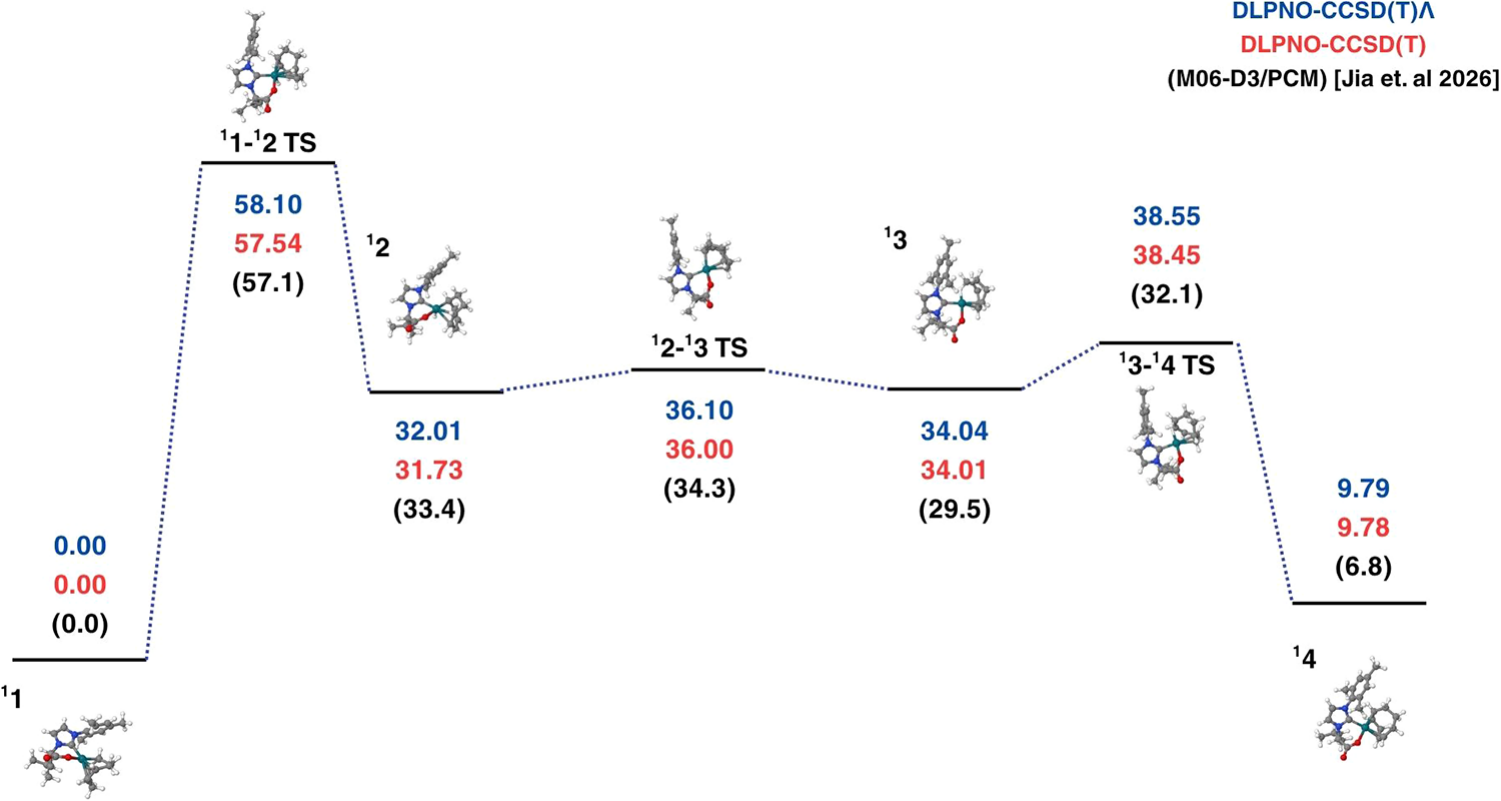
Comparison of DLPNO–CCSD­(T)_Λ_ and DLPNO–CCSD­(T) energetics for the different configurations of the (NHC)­Rh­(Cod) complex, as reported by Jia et al.^113^ Each configuration, including minima and transition states, is labeled above.

### VI.E L7 Large Dimers

Another application of DLPNO–CCSD­(T)_Λ_ on larger molecules is the L7 test set of Sedlak, Pulay, Hobza, and co-workers.^114^ These molecules are of particular interest due to the discrepancies between CCSD­(T) and FN-DMC for some of the larger molecules.^115–118^ In Table [Table tblIV], we present counterpoise-corrected DLPNO–CCSD­(T)_Λ_ and DLPNO–CCSD­(T) interaction energies with the cc-pVDZ basis for five of the dimers in the L7 test set. NormalPNO was used in this study, though we expect the results to be similar to TightPNO since all pairs are strong pairs. The results show that DLPNO–CCSD­(T)_Λ_ and DLPNO–CCSD­(T) do not provide significantly different results for interaction energies, all with errors less than 0.1 kcal mol^–1^. This is true even for the coronene dimer as well as the two systems containing circumcoronene, for which FN-DMC and CCSD­(T) results differ by at least 1.2 kcal mol^–1^.^116^ The agreement of DLPNO–CCSD­(T) and DLPNO–CCSD­(T)_Λ_ results are encouraging, though further studies are required to properly account for other potential shortcomings of CCSD­(T), such as contributions from higher-order coupled-cluster excitations.

**IV tblIV:** Counterpoise-Corrected L7 ^114^ Dimer Interaction Energies, Evaluated with Both DLPNO–CCSD­(T)_Λ_ and DLPNO–CCSD­(T) at the cc-pVDZ Basis with NormalPNO Convergence[Table-fn tIVfn1]

Interaction	*N*_atom_ (nbf)	Δ*E*_DLPNO–CCSD(T)_Λ__	Δ*E*_DLPNO–CCSD(T)_
Coronene Dimer PD (C2C2PD)	72 (792)	–9.05	–9.08
Circumcoronene Adenine Stack (C3A)	87 (1011)	–7.86	–7.90
Circumcoronene/Guanine-Cytosine Stack (C3GC)	101 (1162)	–11.67	–11.68
Stacked Octadecane Dimer (CBH)	112 (884)	–5.24	–5.30
Guanine-Cytosine Stack (GCGC)	58 (792)	–8.85	–8.84
Stacked Guanine Trimer (GGG)	48 (537)	+1.01	+1.04
Phenylalanine Trimer (PHE)	87 (840)	–18.08	–18.16

a The number of atoms (*N*_atom_) and basis functions (nbf) are also included. All energetics are reported in kcal mol^–1^.

### VI.F Cope Rearrangement

As a final benchmark of our code, we present results for the barrier height of the Cope Rearrangement of semibullvalene (C_8_H_8_), and bullvalene (C_10_H_10_). Canonical CCSDT­(*Q*) computations have been performed for semibullvalene/cc-pVDZ­(p,p) in ref 68. cc-pVDZ­(p,p) represents a modified Dunning cc-pVDZ basis, where only polarization functions up to p orbitals are used for heavy atoms. In Table [Table tblV], we present results for the barrier heights for semibullvalene and bullvalene, in kcal mol^–1^, comparing our DLPNO–CCSD­(T), CCSD­(T)_Λ_, CCSDT, and CCSDT­(*Q*) algorithms. For semibullvalene, our results suggest a *T*-(*T*) increment of 0.116 kcal mol^–1^ (vs 0.139 kcal mol^–1^ computed in ref 68), and a (*Q*) increment of −0.265 kcal mol^–1^ (vs −0.268 kcal mol^–1^ from ref 68). These error bars are acceptable, being within 0.03 kcal mol^–1^ for the triples increment, and 0.01 kcal mol^–1^ for the quadruples contribution. Thus, we also run computations for bullvalene, using our DLPNO values as a proxy for the performance of (*T*) vs (*T*)_Λ_ vs CCSDT. These results suggest that in this case, (*T*)_Λ_ corrects for the error in the direction of *T*-(*T*), but appears to overshoot the correction, allowing (*T*) to perform better than (*T*)_Λ_ relative to CCSDT. In addition, the (*Q*) correction also makes (*T*) look more attractive, correcting in the opposing direction of the *T*-(*T*) increment. This gives evidence to support that the success of (*T*) results largely from error cancellation, and though (*T*)_Λ_ performs worse than (*T*) in this instance; the errors are still acceptable (being on the order of around 0.3 kcal mol^–1^). Based on all the systems we studied thus far, it seems like the (*T*)_Λ_ – (*T*) difference can be used as a diagnostic to test the reliability of the (*T*) correction, where small differences, like for noncovalent interactions, suggest that (*T*) is reliable. Large errors (on the order of 1 kcal mol^–1^ or more) would suggest that the (*T*) correction would not be reliable.

**V tblV:** Comparing DLPNO–CCSD­(T), CCSD­(T)_Λ_, CCSDT, and CCSDT­(*Q*) Barrier Heights for the Cope Rearrangement of Semibullvalene and Bullvalene, cc-pVDZ­(p,p) Basis, Presented in kcal mol^–1^

Reaction	Δ*E*_DLPNO–CCSD(T)_	Δ*E*_DLPNO–CCSD(T)_Λ__	Δ*E*_DLPNO–CCSDT_	Δ*E*_DLPNO–CCSDT(*Q*)_
Semibullvalene (C_8_H_8_)	+6.693	+7.073	+6.809	+6.545
Bullvalene (C_10_H_10_)	+13.841	+14.262	+14.012	+13.680

## VII Conclusion

In this work, we present lambda equations for the *t*_1_-transformed DLPNO–CCSD method in our prior work^82^ and in the freely available Psi4 quantum chemistry package.^102^ The errors for relative energies with respect to canonical CCSD­(T)_Λ_ are typically on the order of 0.1–0.2 kcal mol^–1^, as evidenced by our test cases with the pericyclic reactions and noncovalent interactions from the S22 data set. We showed that our algorithm provides qualitatively correct answers relative to CCSDT at points of failure for CCSD­(T) on potential energy surfaces, such as the CH_3_I + OH^–^ *S*_*N*_2 potential energy surface. In those extreme cases, the local correlation error is up to 1 kcal mol^–1^. We also showcase possible applications of our linear-scaling algorithm to larger systems, such as the rhodium catalyst complex (66 atoms), and larger noncovalent dimers in the L7 data set (up to 112 atoms). This code is publicly accessible on a developmental branch of Psi4, and will be made available in a future release. In the future, we plan to apply this code to larger multireference systems, as well as develop lambda equations for higher orders of local coupled-cluster theory, such as CCSDT,^83^ and corresponding asymmetric corrections such as CCSDT­(Q)_Λ_.

## Supplementary Material



## Data Availability

The data that supports the findings of this study are available with the article and its Supporting Information. The Psi4 source code for DLPNO–CCSD­(T)_Λ_ can be found at https://github.com/zizitoth/psi4/tree/dlpno_lambda

## Appendix A: Dipole Moments

Though dipole moments are not presented in our work, expressions for computing the DLPNO–CCSD contribution to the molecular dipole moment using our formalism are presented below, as currently available in our code. The DLPNO–CCSD (non-HF) contribution to the dipole moment can be written in terms of the one-particle density matrix, given in terms of the *x* coordinate. There is an analogous expression for *y* and *z*.

A1
$$\left\langle {\mathrm{\mu ̂}}_{x}\right\rangle =D_{\mathrm{ij}}\left\langle i\right|\mathrm{x̂}\left|j\right\rangle +\left(D_{i}^{a_{\mathrm{ii}}}+\lambda_{i}^{a_{\mathrm{ii}}}\right)\left\langle i\right|\mathrm{x̂}\left|a_{\mathrm{ii}}\right\rangle +\sum_{i,j}D_{a_{\mathrm{ij}}b_{\mathrm{ij}}}^{\mathrm{VV}}\left\langle a_{\mathrm{ij}}\right|\mathrm{x̂}\left|b_{\mathrm{ij}}\right\rangle +\sum_{i}D_{a_{\mathrm{ii}}b_{\mathrm{ii}}}^{V}\left\langle a_{\mathrm{ii}}\right|\mathrm{x̂}\left|b_{\mathrm{ii}}\right\rangle$$

with the different components of the one-particle density matrix (OPDM) being

A2
$$D_{\mathrm{ij}}=-\rho_{\mathrm{ij}}^{\mathrm{OO}}-\left(\lambda_{i}^{e_{\mathrm{ii}}}S_{e_{\mathrm{jj}}}^{e_{\mathrm{ii}}}T_{j}^{e_{\mathrm{jj}}}\right)$$

A3
$$D_{i}^{a_{\mathrm{ii}}}=2T_{i}^{a_{\mathrm{ii}}}+\left(S_{a_{\mathrm{ij}}}^{a_{\mathrm{ii}}}U_{\mathrm{ij}}^{a_{\mathrm{ij}}e_{\mathrm{ij}}}S_{e_{\mathrm{jj}}}^{e_{\mathrm{ij}}}-S_{a_{\mathrm{jj}}}^{a_{\mathrm{ii}}}T_{j}^{a_{\mathrm{jj}}}T_{i}^{e_{\mathrm{ii}}}S_{e_{\mathrm{jj}}}^{e_{\mathrm{ii}}}\right)\lambda_{j}^{e_{\mathrm{jj}}}-\left(S_{a_{\mathrm{jj}}}^{a_{\mathrm{ii}}}T_{j}^{a_{\mathrm{jj}}}\right)\rho_{\mathrm{ji}}^{\mathrm{OO}}-\left(T_{i}^{e_{\mathrm{ii}}}S_{e_{\mathrm{mn}}}^{e_{\mathrm{ii}}}\right)\rho_{e_{\mathrm{mn}}a_{\mathrm{mn}}}^{\mathrm{VV}}S_{a_{\mathrm{ii}}}^{a_{\mathrm{mn}}}$$

A4
$$D_{a_{\mathrm{ij}}b_{\mathrm{ij}}}^{\mathrm{VV}}=\rho_{a_{\mathrm{ij}}b_{\mathrm{ij}}}^{\mathrm{VV}}$$

A5
$$D_{a_{\mathrm{ii}}b_{\mathrm{ii}}}^{V}=\lambda_{i}^{a_{\mathrm{ii}}}T_{i}^{b_{\mathrm{ii}}}$$

## Notes

Geometries for systems studied, as well as absolute energies (ZIP)
